# Role of the central lysine cluster and scrapie templating in the transmissibility of synthetic prion protein aggregates

**DOI:** 10.1371/journal.ppat.1006623

**Published:** 2017-09-14

**Authors:** Bradley R. Groveman, Gregory J. Raymond, Katrina J. Campbell, Brent Race, Lynne D. Raymond, Andrew G. Hughson, Christina D. Orrú, Allison Kraus, Katie Phillips, Byron Caughey

**Affiliations:** Laboratory of Persistent Viral Diseases, Rocky Mountain Laboratories, National Institute of Allergy and Infectious Diseases, National Institutes of Health, Hamilton, Montana, United States of America; Dartmouth Medical School, USA, UNITED STATES

## Abstract

Mammalian prion structures and replication mechanisms are poorly understood. Most synthetic recombinant prion protein (rPrP) amyloids prepared without cofactors are non-infectious or much less infectious than *bona fide* tissue-derived PrP^Sc^. This effect has been associated with differences in folding of the aggregates, manifested in part by reduced solvent exclusion and protease-resistance in rPrP amyloids, especially within residues ~90–160. Substitution of 4 lysines within residues 101–110 of rPrP (central lysine cluster) with alanines (K_4_A) or asparagines (K_4_N) allows formation of aggregates with extended proteinase K (PK) resistant cores reminiscent of PrP^Sc^, particularly when seeded with PrP^Sc^. Here we have compared the infectivity of rPrP aggregates made with K_4_N, K_4_A or wild-type (WT) rPrP, after seeding with scrapie brain homogenate (ScBH) or normal brain homogenate (NBH). None of these preparations caused clinical disease on first passage into rodents. However, the ScBH-seeded fibrils (only) led to a subclinical pathogenesis as indicated by increases in prion seeding activity, neuropathology, and abnormal PrP in the brain. Seeding activities usually accumulated to much higher levels in animals inoculated with ScBH-seeded fibrils made with the K_4_N, rather than WT, rPrP molecules. Brain homogenates from subclinical animals induced clinical disease on second passage into “hamsterized” Tg7 mice, with shorter incubation times in animals inoculated with ScBH-seeded K_4_N rPrP fibrils. On second passage from animals inoculated with ScBH-seeded WT fibrils, we detected an additional PK resistant PrP fragment that was similar to that of *bona fide* PrP^Sc^. Together these data indicate that both the central lysine cluster and scrapie seeding of rPrP aggregates influence the induction of PrP misfolding, neuropathology and clinical manifestations upon passage *in vivo*. We confirm that some rPrP aggregates can initiate further aggregation without typical pathogenesis *in vivo*. We also provide evidence that there is little, if any, biohazard associated with routine RT-QuIC assays.

## Author summary

Differences in the folding and packing of the prion protein into aggregates are thought to be the molecular basis for variability in prion transmissibility. Recombinant prion protein aggregates prepared without non-protein cofactors typically are less tightly packed within residues ~90–160 and are much less infectious than *bona fide* tissue-derived prions. Substitution of a central cluster of 4 positively charged lysines within residues 101–110 of the prion protein with uncharged residues allows the formation of synthetic recombinant prion aggregates with larger tightly packed cores. Inoculation of these mutant and wild-type synthetic aggregates into animals demonstrated that both the central lysine cluster as well as templating with *bona fide* prions, strongly influence both the initiation of self-propagating transmissible misfolding of the endogenous prion protein and the resultant neuropathological and clinical outcomes. The results also confirm that some recombinant prion protein aggregates can replicate *in vivo* without causing classical prion disease pathogenesis.

## Introduction

Prion diseases, or transmissible spongiform encephalopathies (TSEs), are fatal neurodegenerative diseases of infectious, genetic, or spontaneous origin. A common feature of these diseases is the misfolding and aggregation of the cellular prion protein (PrP^C^) into TSE-associated (Scrapie, or PrP^Sc^) forms. PrP^Sc^ is often resistant to digestion by proteinase K (PK) and, as such, may also be called PrP^Res^. PK digestion typically leaves a resistant core of residues ~90–231, attributed to ordered aggregation and the transformation of PrP^C^’s alpha-helical and natively disordered regions into tightly packed assemblies with high beta-sheet content [1, 2]. Conversion of PrP^C^ to PrP^Sc^ is driven by a templated seeded polymerization mechanism in which PrP^Sc^ recruits and catalyzes the refolding of PrP^C^ into growing PrP^Sc^ aggregates [3–7].

Since the inception of the hypothesis that an altered form of prion protein is the infectious agent of TSEs, researchers have attempted to produce prion infectivity from PrP molecules alone under chemically defined conditions *in vitro*. Various *in vitro* conversion reactions have been developed [4, 8–18]. These techniques exploit the self-replicating ability of PrP^Sc^ using natural PrP^C^ or similarly soluble and protease-sensitive PrP (PrP^Sen^) substrates from various sources. PrP^C^ substrates for these reactions can be contained in crude brain homogenates [8] or cell lysates [19], or be purified from various sources [4, 20]. In addition, recombinant PrP^Sen^ (rPrP^Sen^) substrates can be expressed and isolated from E. coli [11, 12, 13, 21]. Sonicated protein misfolding cyclic amplification (PMCA) reactions using brain homogenate as a source of PrP^C^ substrate can recapitulate many attributes of the initial PrP^Sc^ seed and generate substantial levels of infectivity [8]. Purified PrP^C^ or rPrP^Sen^ substrates have also been converted by PMCA to infectious forms on their own [14], but the generation of high-titered rPrP prions that more fully recapitulate the properties of the initial PrP^Sc^ seed has required the presence of cofactors such as RNA and/or phospholipids [21–25].

Prion-seeded polymerization of rPrP^Sen^ also occurs in shaken reactions in the absence of cofactors or denaturing conditions at near neutral pH, hereafter referred to as real time quaking-induced conversion (RT-QuIC) conditions [16, 17]. Sano et al [26] demonstrated that some strain properties can be conserved transiently in recombinant PrP^Res^ (rPrP^Res^) generated under RT-QuIC conditions, but with low infectivity. rPrP^Res^ generated under such conditions has many characteristics of infectious PrP^Sc^ including partial PK-resistance, seeding capabilities, and high beta-sheet secondary structure. However, these conversion products tend to have smaller PK-resistant cores comprised of relatively low molecular weight C-terminal fragments of ~8–12 kDa from the region of residues ~160–231 [27, 28]. To date there have been no reports of robust infectivity from rPrP^Res^ generated under these, or similar, non-denaturing conditions at neutral pH and without cofactors. The Sano study showed a modest increase in infectivity in a single round of RT-QuIC amplification; however, the infectivity was lost in subsequent serial RT-QuIC reactions [26]. The authors attribute this to rapid growth of off-pathway aggregates that overtake the faithful templating activity of the original prion seeds.

Reduced specific infectivity in certain rPrP amyloid preparations has been attributed to incomplete refolding of residues 90-~160, which are more tightly packed and highly protease resistant in more infectious forms of PrP^Sc^ [9, 14, 15, 29, 30]. Tight packing of the 90-~160 region in the absence of cofactors is impeded by a highly conserved cluster of four lysines within residues 101–110 (the central lysine cluster or CLC) [28, 31–33]. We have hypothesized that this is due to repulsion of their cationic side chains in the absence of ion pairing with suitable anionic residues, cofactors or salts. In any case, wild-type rPrP amyloids formed without cofactors tend to have PK-resistant cores that are restricted to C-terminal residues ~160–231 [28, 32] Mutations of the 4 lysines to alanines (K_4_A) or asparagines (K_4_N), which neutralize the lysines within the CLC, allow formation of RT-QuIC products with N-terminally extended PK-resistant cores and with infrared spectra more reminiscent of *bona fide* PrP^Sc^ in terms of β-sheet amide I vibrations [32, 33]. In this study, we have tested whether these scrapie-seeded RT-QuIC conversion products are infectious for rodents. Furthermore, we tested the influence of CLC mutations on the resulting disease phenotypes.

## Results

### PK-resistant cores of conversion products differ with scrapie seeding and K_4_N mutation

**Fig 1:**
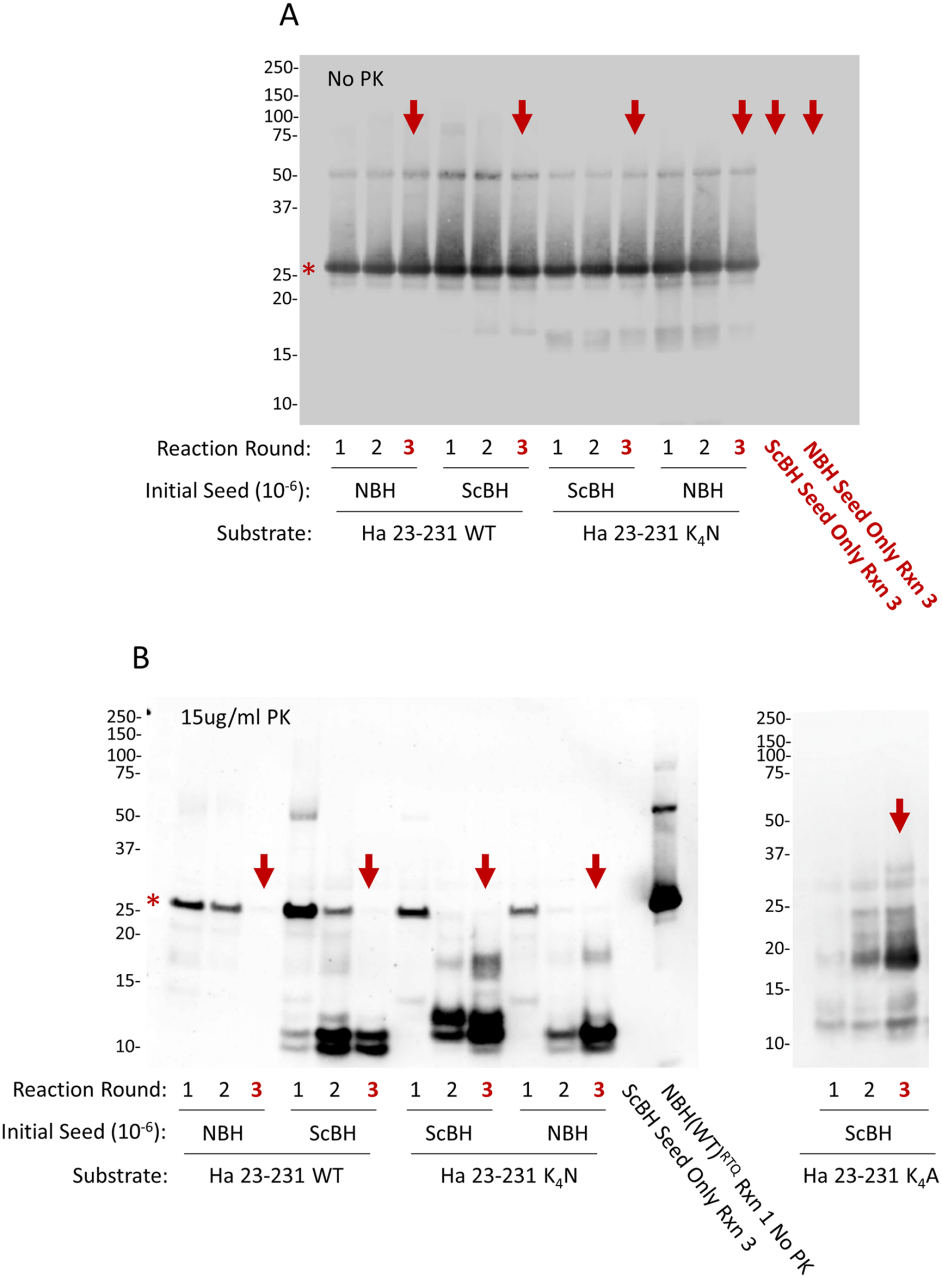
Western blot analysis of multi-round RT-QuIC conversion products. Immunoblot detection without (A) or with (B) proteinase K digestion using polyclonal R20 antiserum directed against C-terminal residues 218–231. Red asterisks indicate undigested rPrP^sen^. Third round reactions (red arrows) were used for animal inoculations. The last lane (B) indicated as NBH(WT)^RTQ^ Rxn 1 No PK, is a representative undigested conversion product from the reaction also shown in lane one in panel A. Similar results were obtained in at least 3 independent multi-round reactions. The appearance of a weak ~17 kDa band in the ScBH(WT) reaction round 2 was only observed in 1 of 9 total reaction rounds and was apparently propagated into round 3. Thus it was not representative of our findings overall.

We generated RT-QuIC conversion products seeded with either scrapie brain homogenate (ScBH) or normal brain homogenate (NBH) using recombinant hamster (residues 23–231) wild-type (WT), K_4_N, or K_4_A rPrP^sen^ as substrates, hereafter to be called ScBH(WT)^RTQ^, ScBH(K_4_N)^RTQ^, ScBH(K_4_A)^RTQ^, NBH(WT)^RTQ^, NBH(K_4_N)^RTQ^ and NBH(K_4_A)^RTQ^ products, respectively. To ensure that the ScBH(WT)^RTQ^, ScBH(K_4_N)^RTQ^, and ScBH(K_4_A)^RTQ^ products to be used for inoculations did not contain residual infectivity from the initial scrapie seed, we performed three serial rounds of tube-based RT-QuIC-like reactions with 1000-fold dilutions between rounds. These rounds diluted the ScBH seed (a 10^−6^ brain tissue dilution) to 10^−12^, a dilution that was far beyond the limit of detection of our rodent bioassays and RT-QuIC [16]. PK-treated and untreated RT-QuIC reaction products from each round were analyzed by Western blot using the polyclonal R20 antiserum directed against C-terminal residues 218–231 (Fig 1). No PrP was detected (Fig 1A, red arrows) in reactions without any rPrP^Sen^ substrate that were seeded initially with 10^−6^ scrapie brain and then subjected to serial RT-QuIC rounds. In the reactions containing rPrP^Sen^ substrate we detected predominant ~10 and 12 kDa C-terminal PK-resistant bands in the ScBH(WT)^RTQ^ products similar to our previous findings [32], whereas the NBH(WT)^RTQ^ product showed no PK-resistant rPrP (Fig 1B). In contrast, both the ScBH(K_4_N)^RTQ^ and NBH(K_4_N)^RTQ^ products contained multiple PK-resistant bands, but the banding profile differed between the two. Whereas the ScBH(K_4_N)^RTQ^ and ScBH(K_4_A)^RTQ^ products had predominant ~12, 13 and 17 kDa bands, the NBH(K_4_N)^RTQ^ products had a predominant 12-kDa band, with much weaker bands at ~10 and 17 kDa, consistent with previous observations (Fig 1B, [32] [33]). Similar Western blot results were obtained in two additional independent 3-round RT-QuIC reactions with the WT, K_4_N and K_4_A rPrP substrates. These results confirmed that both scrapie seeding and the CLC mutation affected the PK-resistant cores of the rPrP aggregates produced with shaking under mild conditions in the absence of cofactors and denaturants.

### Propagation of prion seeding activity in animals inoculated with scrapie-seeded RT-QuIC products

The various RT-QuIC conversion products were inoculated intracerebrally into hamsters or Tg7 transgenic mice that over-express hamster PrP^C^ [34]. We will call these first-passage (P1) animals ScBH(WT)^P1^, ScBH(K_4_N)^P1^, NBH(WT)^P1^ and NBH(K_4_N)^P1^ according to their inoculum. No clinical signs of prion disease were seen by blinded observers within 652 and 492 days post inoculation (dpi) in any of the P1 hamsters or mice, respectively (Table 1 and S1A & S1B Table). To investigate the possibility of subclinical pathology we collected brains at various time points for RT-QuIC, immunoblotting, and histopathological analyses. Moreover, animals that became sick or injured without apparent outward signs of prion disease were also collected and analyzed when possible.

**Table 1:** Summary of results from P1 animals.

INOCULA	CLINICAL^1^		RT-QUIC^1^		WESTERN BLOT^1^		HISTOLOGY^1^	
	Ha^2^	Tg7 Mice	Ha	Tg7 Mice	Ha	Tg7 Mice	Ha	Tg7 Mice
NBH(WT)^RTQ^ 1:1	0/6^#^	0/4	0/6	0/4	0/1	0/1	0/4	0/4
NBH(WT)^RTQ^ 1:10	0/6	0/4	0/6	0/4	0/1	0/1	0/6	0/4
NBH(K_4_N)^RTQ^ 1:1	0/6	0/4	0/5	0/4	0/1	0/1	0/3	0/4
NBH(K_4_N)^RTQ^ 1:10	0/6	0/6	0/5	0/6	0/1	0/1	0/5	0/2
ScBH(WT)^RTQ^ 1:1	0/6	0/6	**6/6**	**6/6**	0/6	**1/6**	0/4	4/6*
ScBH(WT)^RTQ^ 1:10	0/6	0/6	**6/6**	**6/6**	0/1	0/6	0/5	0/6
ScBH(K_4_N)^RTQ^ 1:1	0/6	0/4	**6/6**	**4/4**	**1/6**	**4/4**	**3/4**	**4/4**
ScBH(K_4_N)^RTQ^ 1:10	0/6	0/2	**6/6**	0/2	0/1	0/1	0/6	0/2
ScBH(K_4_A)^RTQ^ 1:1	0/5	NA	**5/5**	NA	0/5	NA	**2/5**	NA
ScBH seed only 1:1	0/6	0/4	0/4	0/4	0/1	0/2	0/2	0/4
NBH seed only 1:1	0/6	NA	0/4	NA	0/1	NA	0/2	NA
Uninoculated	0/4	NA	0/4	NA	0/3	NA	0/1	NA

^#^number positive/total animals surveyed;

*Three animals showed mild astrogliosis but no other histopathology;

^1^Animals were scored positive based on the criteria outlined in the methods;

^2^Hamsters

First, we used end-point dilution RT-QuIC [16] to measure the prion seeding activity in the brains of the animals. Neither the wild-type nor mutant rPrP products of reactions seeded with NBH (i.e., NBH(WT)^RTQ^ and NBH(K_4_N)^RTQ^) elicited any detectable seeding activity after either >652 or 492 d incubations in the brains of P1 hamsters or Tg7 mice, respectively (S1A & S1B Table). This was not surprising in the case of the NBH(WT)^P1^ animals because there was no apparent rPrP^Res^ or ThT-positive amyloid in the inoculated NBH(WT)^RTQ^ preparation (Fig 1 and S1 Fig). However, in the case of NBH(K_4_N)^P1^ animals, the inoculum contained partially PK-resistant products. Additionally, both the NBH(K_4_N)^RTQ^ product (Fig 1 and S1 Fig) and the brains from the Tg7 NBH(K_4_N)^P1^ mice collected 2 h after inoculation (n = 2) had RT-QuIC seeding activity, but that activity was not detected in brains collected at later times post-inoculation (S1 Table). Thus, the NBH(K_4_N)^RTQ^ seeding activity failed to be retained or propagate using endogenous PrP^C^ *in vivo*.

**Fig 2:**
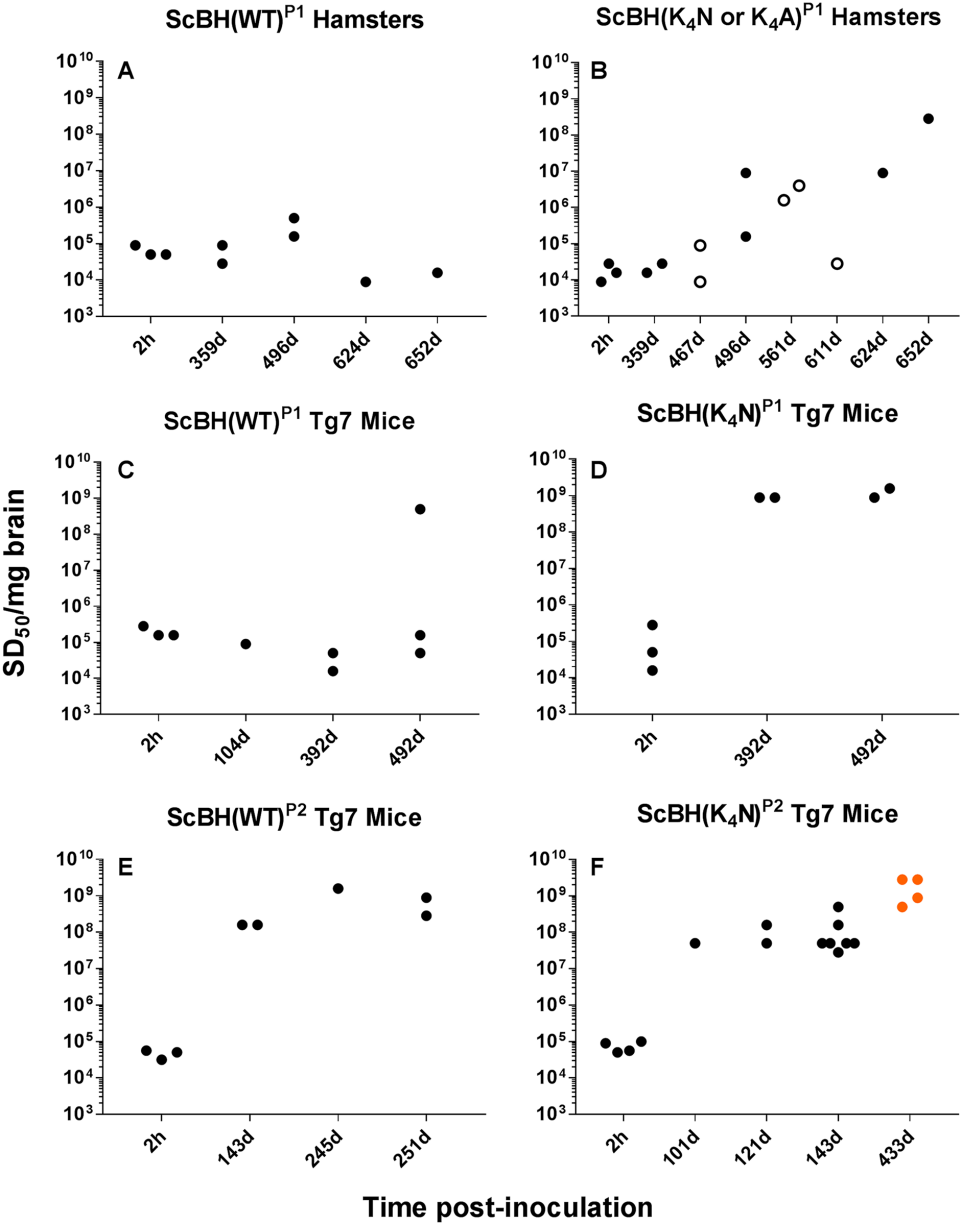
Seeding activities in the brains of inoculated animals. End-point dilution RT-QuIC analysis of brain samples from hamsters and Tg7 mice inoculated with RT-QuIC conversion products. Open circles indicate ScBH(K_4_A)^P1^ hamsters. Orange dots indicate animals with prolonged disease phenotype.

In contrast, seeding activity was detectable in the brains of P1 animals inoculated with *in vitro* products seeded with ScBH (i.e. ScBH(WT)^RTQ^ and ScBH(K_4_N)^RTQ^) (Table 1, S2 Fig and S1A & S1B Table). In the case of the ScBH(WT)^RTQ^ inoculum, seeding activity was detected in all ScBH(WT)^P1^ animals at >350 dpi, but none of the 6 hamsters (Fig 2A) and only 1 out of 12 Tg7 mice (Fig 2C) showed more than a 10-fold increase in seeding activity over the levels measured in a control group 2 h after inoculation. Much stronger evidence for propagation of seeding activity was observed after inoculations with the ScBH(K_4_N)^RTQ^ product, with 3 out of 4 hamsters (Fig 2B) and all 4 Tg7 mice (Fig 2D) harvested after ≥392 days showing increases in seeding activity of ~500–100,000 fold. Reinforcing the effects of the CLC mutations, we also saw persistence of seeding activity in all 5 hamsters inoculated with an ScBH(K_4_A)^RTQ^ product and increased levels of seeding activity in 2 out of 5 of those animals (S1A Table). The observed effects were dose-dependent because the seeding activity was generally weaker in the brains of the animals inoculated with a 1:10 dilution of the ScBH seeded conversion products in inoculation buffer compared with those diluted 1:1 (S1A & S1B Table). Altogether, these results indicated that both scrapie seeding and the CLC mutations enhanced the *in vivo* propagation capacities of products generated *in vitro* in the absence of cofactors or denaturing conditions.

### Western blot detection of PrP^Res^ in brains of ScBH(K_4_N)^P1^ and ScBH(WT)^P1^ animals

**Fig 3:**
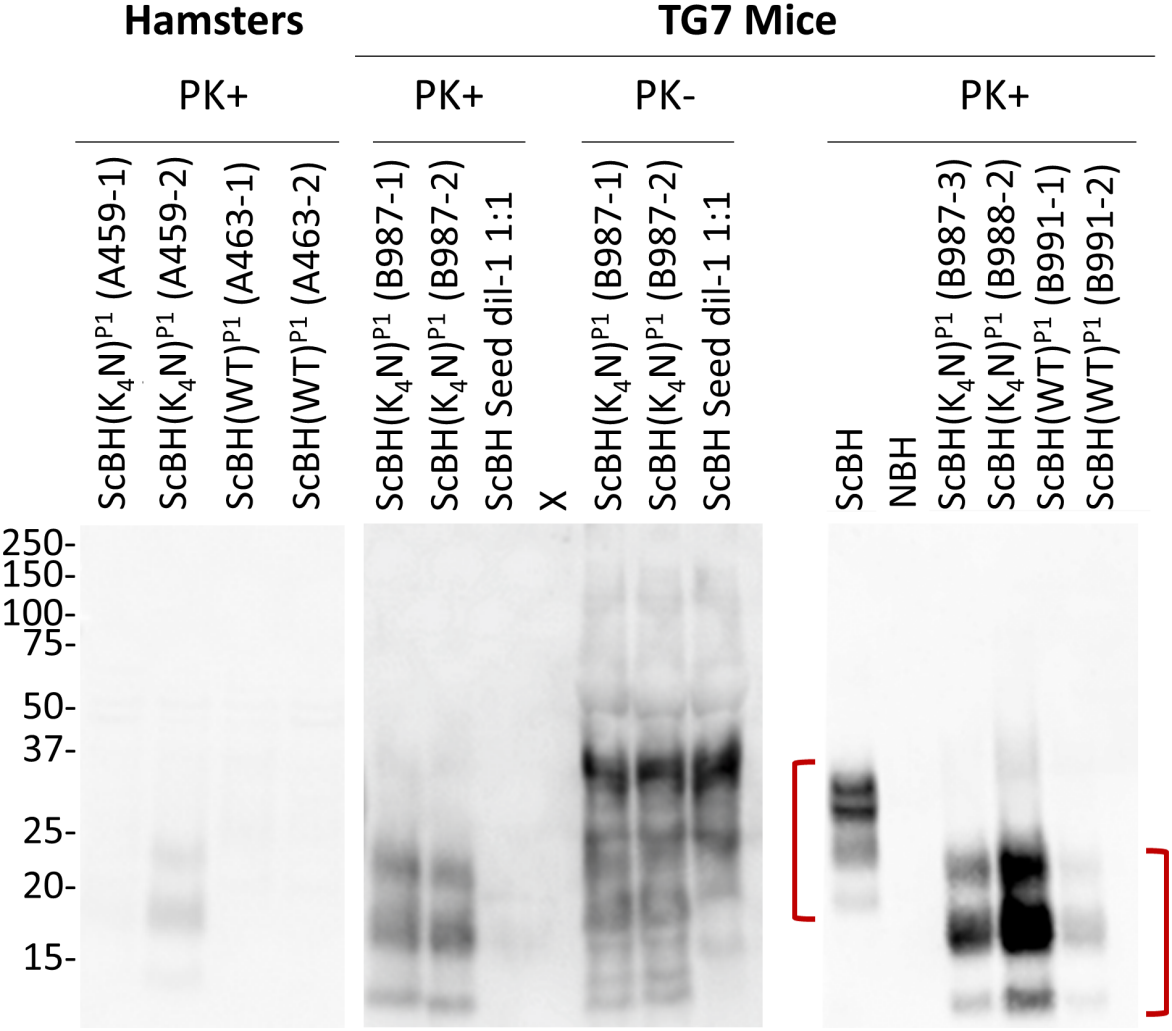
PK-resistant PrP detected in the brains of inoculated animals. Representative Western blots detecting new PrP^Res^ in brains from ScBH(WT or K_4_N)^P1^ hamsters and Tg7 mice using the C-terminal R20 antiserum. Red brackets highlight the differences between the 263K scrapie brain PrP^Res^ and PrP^Res^ in the brains of the RT-QuIC product inoculated animals. Panels were cut and aligned from larger blots that all contained pertinent positive and negative controls to emphasize relevant lanes. “X” indicates a blank lane.

**Fig 4:**
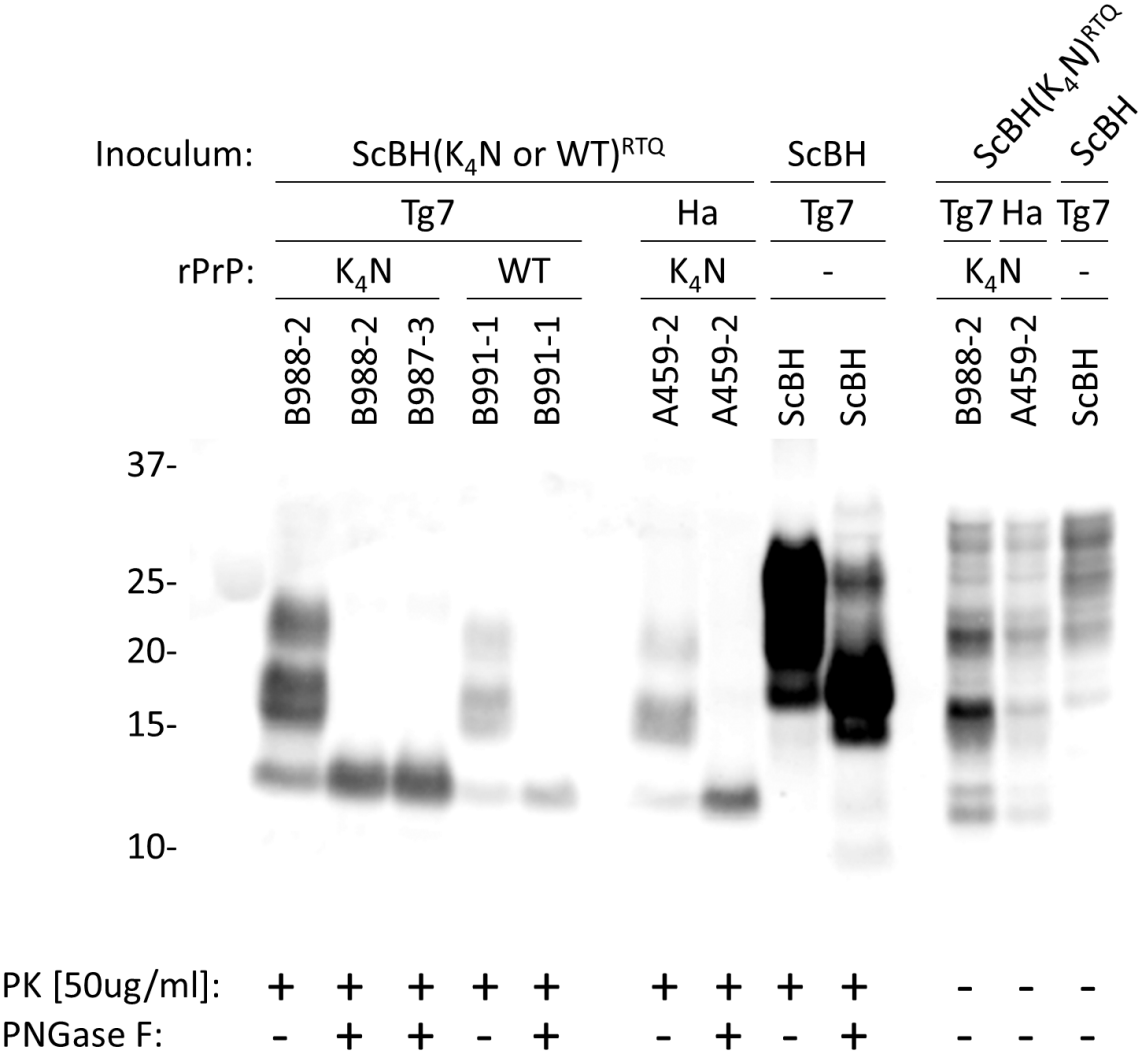
Banding patterns of PrP^Res^ from inoculated animals. Western blot of brain samples from ScBH(WT or K_4_N)^P1^ Tg7 mice and hamsters using R20 antiserum (epitope: residues 218–231). Brain homogenates from ScBH(WT or K_4_N)^P1^ and controls were treated with PK and/or PNGase F prior to immunoblotting as designated.

Prompted by RT-QuIC detection of prion seeding activity in the brains of ScBH(WT)^P1^ and ScBH(K_4_N)^P1^ animals, we analyzed P1 brain tissue for the presence of PrP^Res^ made from endogenous PrP^C^ using a C-terminal PrP antiserum (R20). None of the ScBH(WT)^P1^ or ScBH(K_4_A)^P1^ hamsters showed any evidence of PrP^Res^ (Fig 3 [A463], Table 1, and S1A Table). However, one of the six ScBH(K_4_N)^P1^ hamsters (A459-2) had a distinct PrP^Res^ banding pattern with three bands at ~14, 17, and 22 kDa (Fig 3 and Table 1). This banding pattern differed from the ~20, 25, and 30 kDa banding pattern of *bona fide* 263K ScBH PrP^Res^ that was used to initially seed these RT-QuIC reactions (Fig 3). Furthermore, all four ScBH(K_4_N)^P1^ Tg7 mice showed the same PK-resistant banding pattern with the same molecular weights of ~14, 17, and 22 kDa (Fig 3 and Table 1). One of the six ScBH(WT)^P1^ mice (B991-1) also showed a similar PrP^Res^ banding profile (Fig 3 Table 1). However, no PrP^Res^ was detected in the uninoculated or seed-only control animals or NBH(WT)^P1^ or NBH(K_4_N)^P1^ animals (Fig 3 and Table 1). Epitope mapping (S3 Fig) of the PrP^Res^ detected in the ScBH(WT)^P1^ and ScBH(K_4_N)^P1^ animals indicated that the PrP^Res^ generated *in vivo* following inoculation with ScBH(WT)^RTQ^ and ScBH(K_4_N)^RTQ^ products had a PK-resistant core that was smaller than that of 263K PrP^Res^, and spanned approximately from the R18 epitope (residues 143–156) (S3D Fig) to the C-terminal R20 epitope (S3E Fig). Deglycosylation with PNGase F showed that the multiple bands containing this PK-resistant core were due to differences in N-linked glycosylation (Fig 4). This confirmed that rather than being residual unglycosylated rPrP inoculum, the PrP^Res^ being detected in these brains was made endogenously from glycosylated host-derived PrP^C^.

### Histopathological evidence of prion transmission

**Fig 5:**
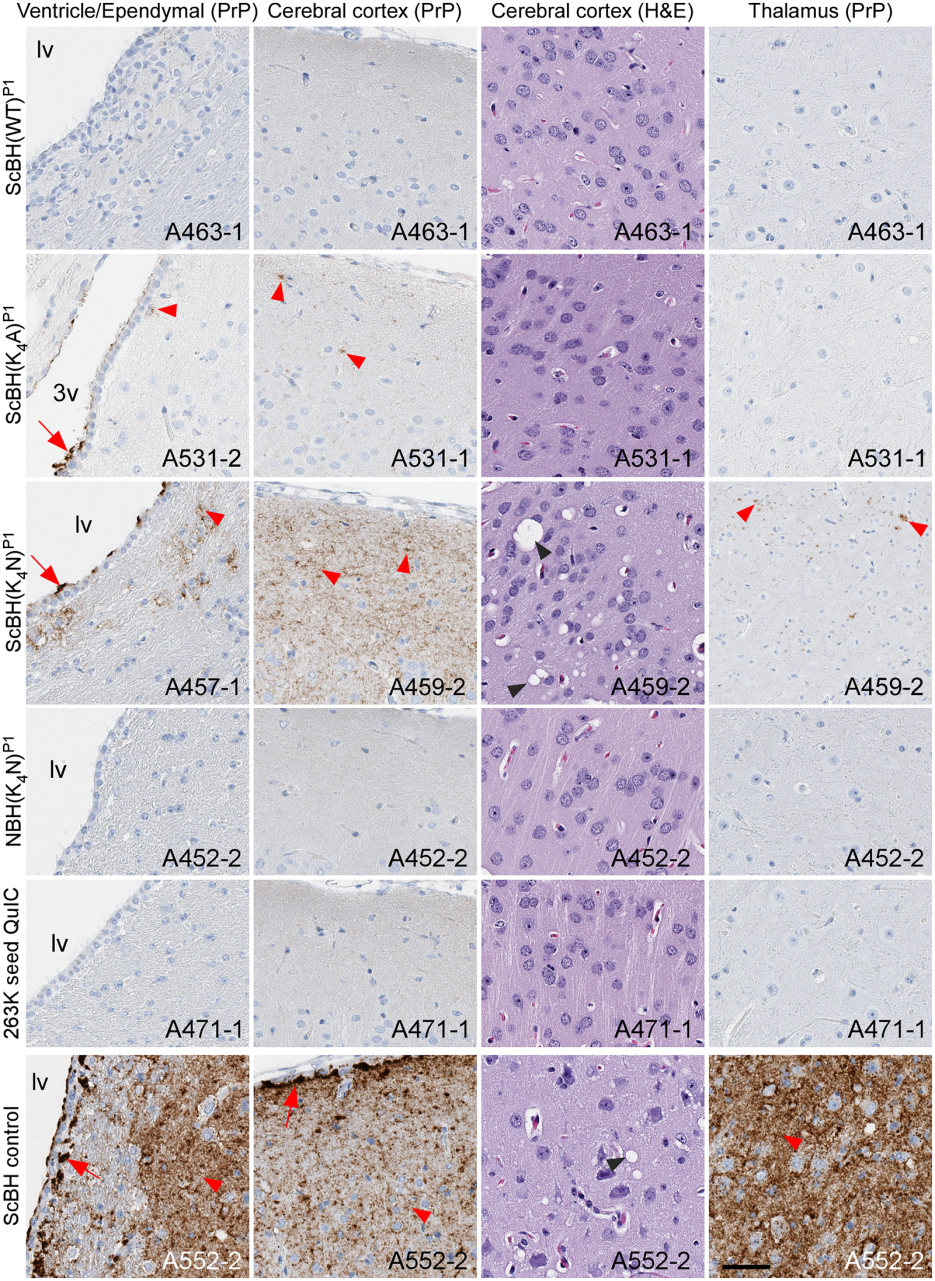
Histopathology of P1 hamsters. Brain regions that show most prominent lesions are displayed. Slides were stained using PrP (EP1802Y) antibody, GFAP antibody, or hematoxylin and eosin. Red arrows indicate punctate aggregates of PrP. Red arrow heads indicate diffuse PrP staining. Black arrow heads indicate spongiosis. *Lv* is lateral ventricle, *3v* is 3^rd^ ventricle. Animal numbers are displayed on the images. Scale bar represents 50 microns.

**Fig 6:**
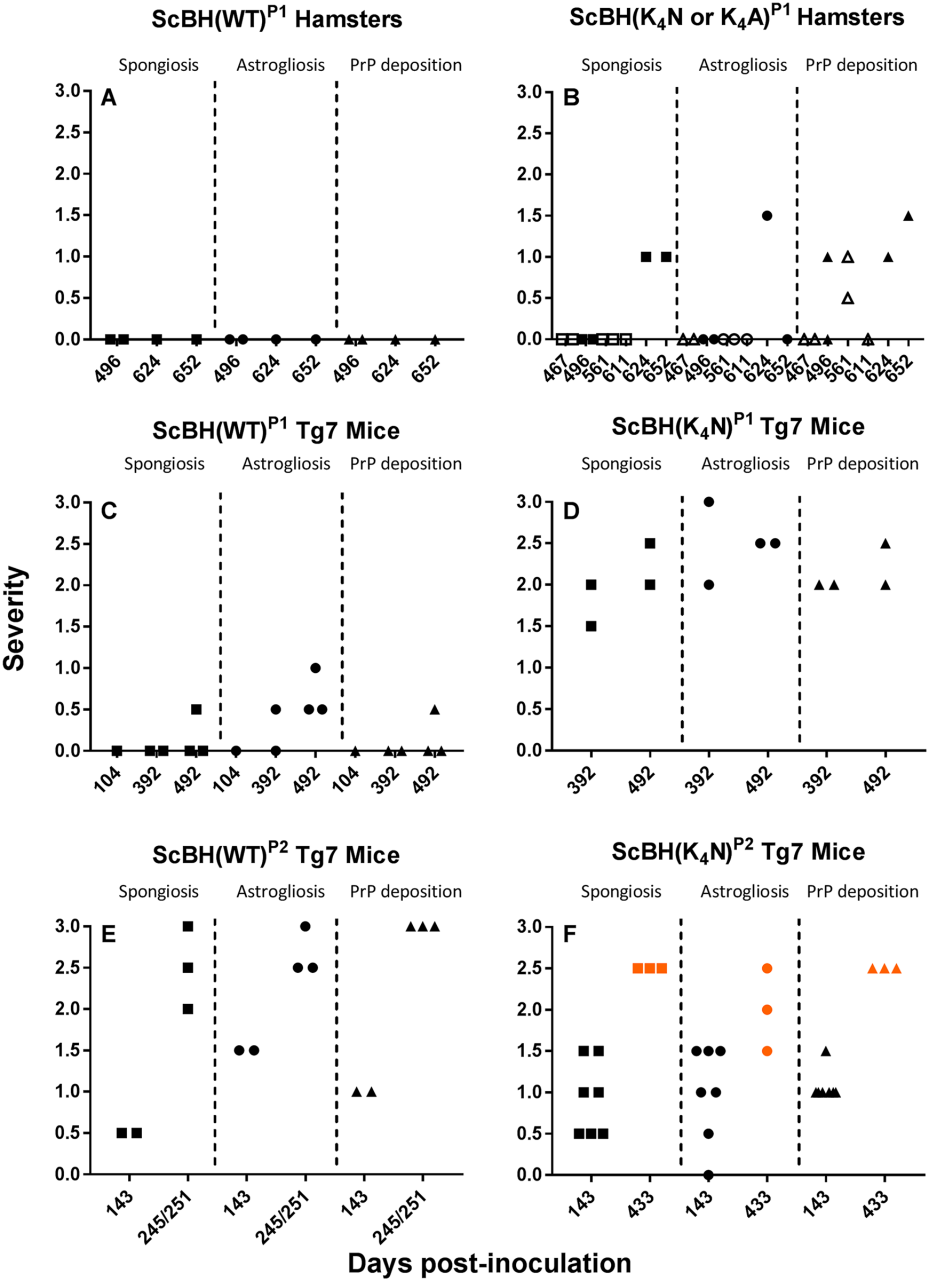
Summary of blinded histopathological analyses of ScBH(WT)^P1^ hamsters (A), ScBH(K_4_N or K_4_A)^P1^ hamsters (B), ScBH(WT)^P1^ Tg7 mice (C), ScBH(K_4_N)^P1^ Tg7 mice (D), ScBH(WT)^P2^ Tg7 mice (E), and ScBH(K_4_N)^P2^ Tg7 mice (F). Spongiosis, astrogliosis, and PrP deposition were ranked for severity and grouped by days post inoculation (DPI). For severity, a value of 0 indicates the absence of the indicated conditions (spongiosis, astrogliosis or PrP deposition), 1 is mild, 2 is moderate, and 3 is severe. Each symbol represents an individual animal. Open symbols indicate ScBH(K_4_A)^P1^ hamsters. Orange symbols indicate animals with prolonged disease phenotype. Details on each animal can be found in S2 Table.

Blinded histopathological analyses of P1 brains were performed to compare astrogliosis (anti-GFAP staining), spongiosis (hematoxylin and eosin staining), and abnormal PrP deposition (anti-PrP antibody EP1802Y staining). The three ScBH(K_4_N)^P1^ hamsters with the highest RT-QuIC seeding activities (≥ ~10^7^ SD_50_/mg brain; S1A Table) displayed mild histolopathological signs of prion disease (Figs 5 and 6, Table 1, S1A and S2 Tables). One hamster (A457-1) had enhanced PrP staining in the lateral ventricle along ependymal cells and adjacent parenchyma near the ventricle but no astrogliosis or spongiosis (S2 Table). Another (A459-2) had widespread diffuse PrP staining and focal vacuolation in the cerebral cortex (Fig 5), but no astrogliosis (S2 Table). A third hamster (A459-1) showed clear PrP^Res^ deposition, mild spongiform change and moderate astrogliosis in the cerebral cortex and hippocampus (Table 1, Fig 6, S1A and S2 Tables). The two ScBH(K_4_A)^P1^ hamsters with the highest seeding activity (≥ ~10^6^ SD_50_/mg brain; S1A Table) had either subtle diffuse PrP^Res^ deposition in the caudal region of the cerebral cortex or plaque-like deposits lining the meninges and ependymal cells. Neither of these hamsters showed signs of astrogliosis or spongiosis (Fig 5, S1A and S2 Tables). None of the ScBH(WT)^P1^ hamsters displayed any histopathology despite having detectable, but relatively low, seeding activity (~10^3^−10^5^ SD_50_/mg brain). Additionally, none of the NBH(WT)^P1^ or NBH(K_4_N)^P1^ hamsters or hamsters inoculated with any of the RT-QuIC products diluted 1:10 showed any histopathological signs of prion disease. Importantly, the neuropathology and PrP^Res^ deposition pattern observed in the three ScBH(K_4_N)^P1^ and two ScBH(K_4_A)^P1^ hamsters, although different from controls, lacked the severity of neuropathology commonly observed in the thalamus of animals inoculated with our standard 263K scrapie prion stock (Fig 5). Paired with our previous observations, the histopathology suggested that laboratory contamination was not an explanation for the unique pathology we observed.

**Fig 7:**
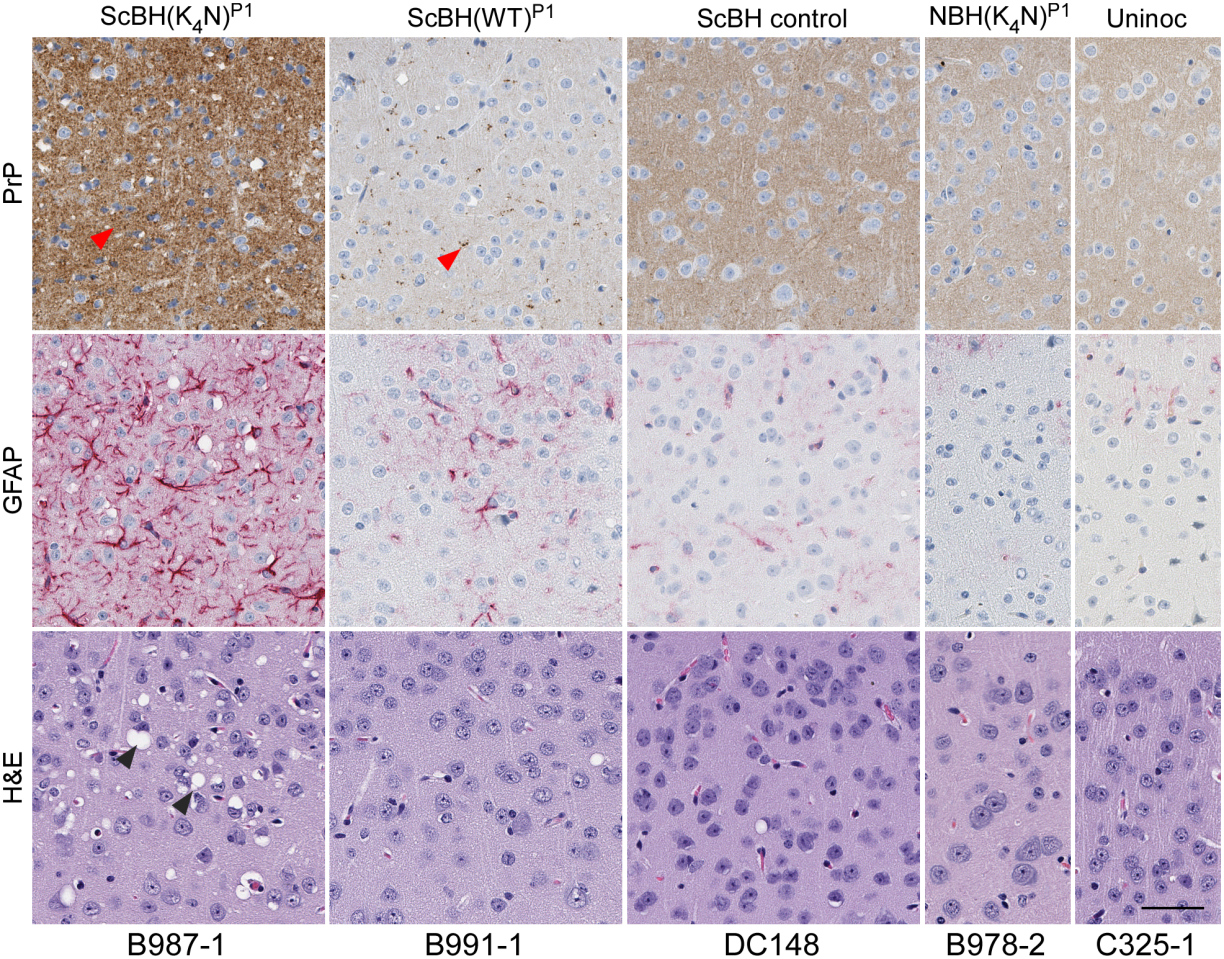
Histopathology of cerebral cortex of Tg7 ScBH(WT or K_4_N)^P1^ mice and controls. Slides were stained using a PrP antibody (EP1802Y), GFAP antibody (for astrocytic activation), or hematoxylin and eosin. Animal numbers are displayed below images. Red arrow heads denote PrP aggregates and black arrows denote vacuolization (spongiosis). Scale bar indicates 50 microns.

More obvious histopathological signs of prion disease were observed in all four of the Tg7 mice inoculated with the ScBH(K_4_N)^RTQ^ product (Figs 6 and 7, S5 Fig, Table 1, S1B and S2 Tables). These mice had prominent spongiform lesions and severe astrogliosis. PrP staining revealed small punctate and granular aggregates in the hypothalamus as well as diffuse punctate staining in the hippocampus, and cortex (S2 Table). One of the mice (B988-1) had additional PrP aggregates in the cerebellum. In contrast to the PrP^Res^ distribution pattern observed in ScBH(K_4_N)^P1^ mice, positive control Tg7 mice inoculated with 263K ScBH had localized neuropathology and PrP^Res^ in the thalamus and brain stem (S4 Fig and S2 Table) and minimal pathology in the cortex and hippocampus (Fig 7 and S5 Fig). Moreover, each of the ScBH(K_4_N)^P1^ mice with histological lesions were also positive for PrP^Res^ by Western blot and each had high (≥ 5x10^8^ SD_50_/mg brain) RT-QuIC seeding activity (S1 Table). One ScBH(WT)^P1^ mouse showed histological signs of prion disease which included small punctate and granular PrP aggregates, along with mild spongiform change and weak astrogliosis (Figs 6 and 7, S5 Fig, Table 1, S1B and S2 Tables). Three additional ScBH(WT)^P1^ mice showed weak, localized astrogliosis but no other histopathological signs of TSE disease. None of the uninoculated mice or the mice inoculated with NBH(WT)^RTQ^, NBH(K_4_N)^RTQ^ or 10-fold dilutions thereof showed any histopathology (Table 1 and S1B Table). Thus, the ScBH(K_4_N)^RTQ^ product elicited neuropathological lesions that were distinct from those of 263K scrapie-inoculated animals and absent in animals receiving the other inocula.

### Second passages (P2) of P1 brain homogenates elicited clinical and biochemical evidence of prion disease

**Fig 8:**
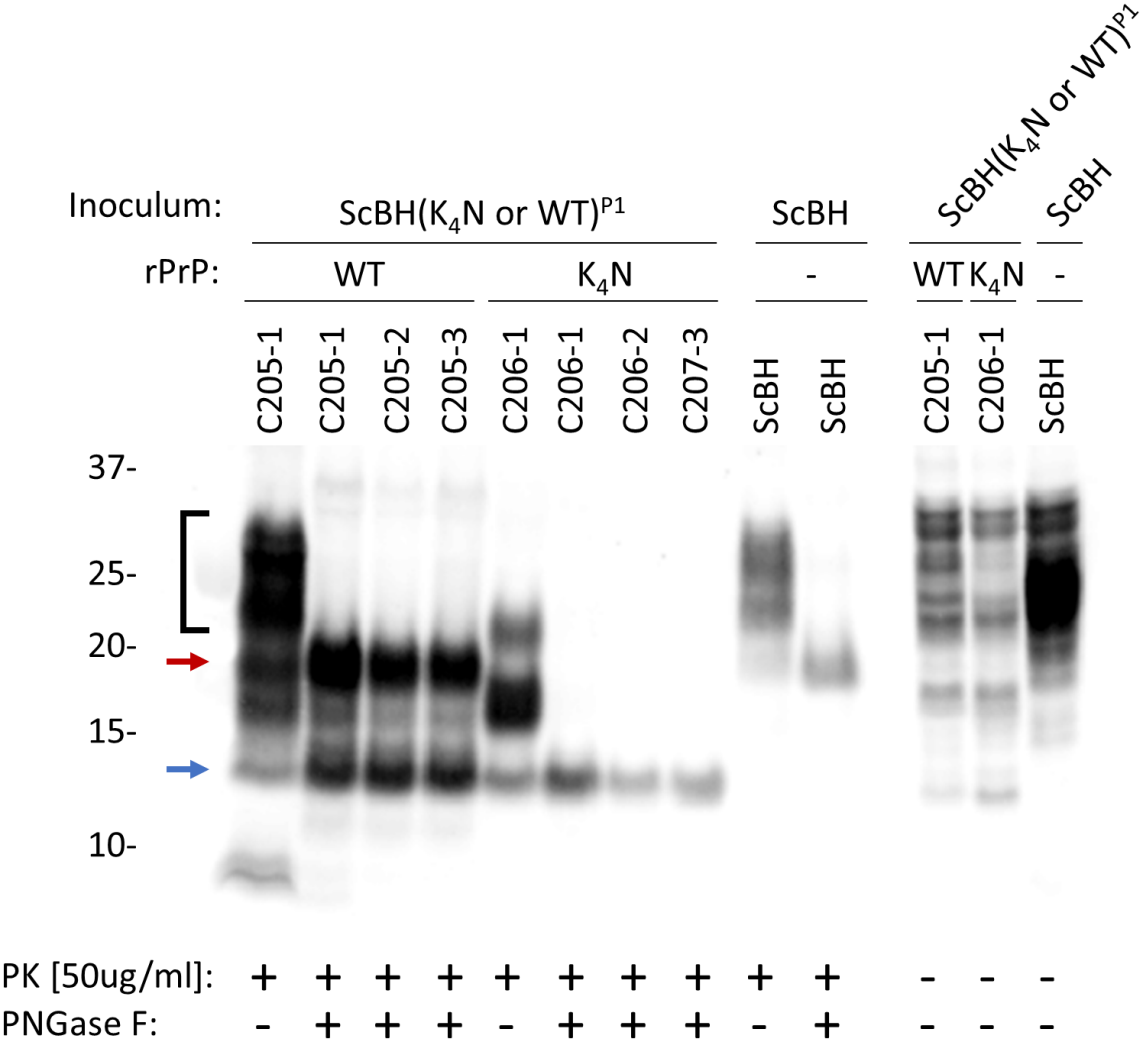
Western blot showing the banding profiles of newly formed PrP^Res^ in brains from ScBH(WT or K_4_N)^P2^ Tg7 mice. PrP^Res^ was detected using R20 antiserum (epitope: 218–231). Brain homogenates from ScBH(WT or K_4_N)^P2^ and controls were treated with PK and/or PNGase F. The ScBH(WT)^P2^ brain homogenates had ~20 and 14 kDa bands (red and blue arrows, respectively), whereas ScBH(K_4_N)^P2^ brain homogenates maintained a single ~14kDa band (blue arrow). ScBH(WT)^P2^ brain homogenate not treated with PNGase F showed higher molecular weight bands (bracket) in addition to those of ScBH(K_4_N)^P2^, reminiscent of the ScBH control.

The transmission properties of P1 brain homogenates was assessed by second passages (P2) into Tg7 mice. Brain homogenates from 3 of the 4 ScBH(K_4_N)^P1^ mice (B987-1, B987-2, and B988-2) and the one ScBH(WT)^P1^ mouse (B991-1) with strong seeding activity and detectable PrP^Res^ were used to inoculate the P2 Tg7 mice (S1C Table). Regarding the ScBH(K_4_N)^P2^ mice, three died without overt clinical signs of disease, one at 101 and two at 121 dpi. However, these mice had unused nestlets, which is a common early manifestation of scrapie disease in mice [35]. At 143 dpi, two additional ScBH(K_4_N)^P2^ mice showed signs of prion disease, including pronounced myoclonus, slight hyperactivity, head tilting, sideways gait, ungroomed coats, and unused nestlets (Table 2 and S1C Table). These and 5 additional non-clinical ScBH(K_4_N)^P2^ mice were euthanized at 143 dpi for further biochemical and histological analysis (see below). Brains from the four remaining ScBH(K_4_N)^P2^ mice were harvested at 433 dpi after prolonged neurological signs including myoclonus, unused nestlets, poor grooming, and jerky movements for >5 months, and, in one case, designation by our standard criteria as having clinical prion disease. All of the ScBH(K_4_N)^P2^ mice, regardless of time of death, had abundant RT-QuIC seeding activity (~10^7^−10^9^ SD_50_/mg tissue; results from representative ScBH(WT)^P2^ and ScBH(K_4_N)^P2^ mice are shown in S6 Fig). However, because the inoculum delivered ~10^5^ SD_50_/mg tissue to the brain, we could not establish that overall seeding activity levels increased post-inoculation by more than ~10,000-fold, with the latter being the accumulation at 433 dpi (Fig 2F). All of the ScBH(K_4_N)^P2^ mice also had PrP^Res^ with ~14, 17, and 22 kDa representing un-, mono- and di-glycosylated bands, respectively (Fig 8, Table 2 and S1C Table), similar to the bands seen in the corresponding P1 mice (Fig 3).

**Table 2:** Summary of results from P2 Tg7 mice.

INOCULA	CLINICAL^1^	RT-QUIC^1^	WESTERN BLOT^1^	HISTOLOGY^1^
	Tg7 Mice	Tg7 Mice	Tg7 Mice	Tg7 Mice
NBH(WT)^P1^	0/13^#^	0/13	0/5	0/13
NBH(K_4_N)^P1^	0/6	0/6	0/5	0/6
ScBH(WT)^P1^	5/5*	**5/5**	**5/5**	**5/5**
ScBH(K_4_N)^P1^	9/14**	**14/14**	**14/14**	**10/10**
Uninoculated	0/4	0/4	0/2	0/4

^1^Animals were scored positive based on the criteria outlined in the methods;

^#^number positive/total animals surveyed;

*2 possible (showing some signs of TSE disease);

** 3 possible and 4 prolonged (over 6 months of subtle signs of TSE disease)

Regarding the ScBH(WT)^P2^ mice, two were harvested at 143 dpi with possible clinical signs. These mice had RT-QuIC seeding activities of SD_50_s ≥5.00x10^7^ (Fig 2E and S1C Table) and PrP^Res^ (Table 2 and S1C Table). The PrP^Res^ in these mice was indistinguishable from that of the ScBH(K_4_N)^P2^ mice shown in Fig 8. At 245–251 dpi the three remaining ScBH(WT)^P2^ mice displayed clinical signs and higher prion seeding activities (S1C Table and Fig 2). Interestingly, immunoblot analysis of brain homogenates of these same mice revealed, in addition to the truncated ~14, 17, and 22 kDa bands seen in the ScBH(K_4_N)^P2^ and 143-dpi ScBH(WT)^P2^ mice (without PNGase F treatment), higher molecular weight bands reminiscent of the ones found in 263K ScBH-inoculated mice (Fig 8; bracket). Following PNGase F treatment of this material we detected two predominant bands: the ~14 kDa band observed in the ScBH(K_4_N)^P2^ mice and a ~20 kDa band similar to 263K PrP^Res^ (Fig 8; blue and red arrows, respectively). Importantly, none of the NBH(WT)^P2^, NBH(K_4_N)^P2^ or uninoculated control mice had any clinical signs or seeding activity in their brains. Furthermore, immunoblot analysis confirmed the absence of PrP^Res^ (Table 2 and S1C Table).

### Histopathological comparisons of second passage mice

**Fig 9:**
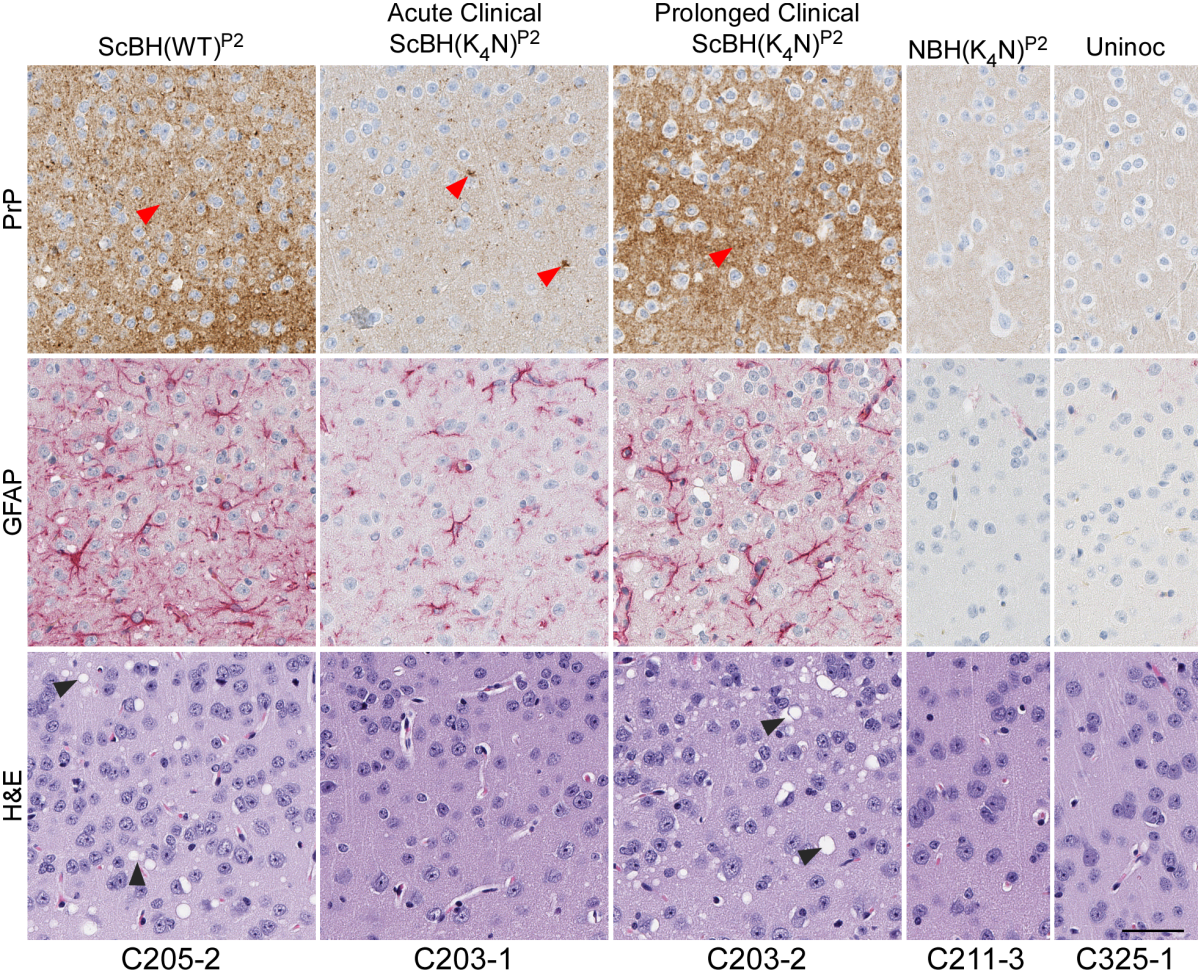
Histopathology of cerebral cortex of Tg7 ScBH(WT or K_4_N)^P2^ mice and controls. A comparison between ScBH(K_4_N)^P2^ Tg7 mice that had acute TSE disease at 143 dpi and ScBH(K_4_N)^P2^ Tg7 mice that had prolonged clinical signs out to 433 dpi is shown. Red arrow heads denote PrP aggregates and black arrow heads denote vacuolization (spongiosis). Slides were stained using a PrP antibody (EP1802Y), GFAP antibody or hematoxylin and eosin. Animal numbers are displayed below images. Scale bar indicates 50 microns.

After second passage, all of the ScBH(K_4_N)^P2^ and ScBH(WT)^P2^ mice showed at least two out of three of the neuropathological lesions examined, namely spongiosis, astrogliosis and PrP deposition but with varied intensity and distribution (Figs 6 and 9, S7 Fig, S1C and S2 Tables). The most consistent differences between the ScBH(K_4_N)^P2^ and ScBH(WT)^P2^ were i) the level of spongiosis at the relatively early 143 day timepoint (Fig 6) and ii) the more focal lesions in the ScBH(K_4_N)^P2^ mice compared to the ScBH(WT)^P2^ mice (S2 Table). The ScBH(K_4_N)^P2^ mice that displayed the prolonged clinical phenotype, as mentioned above, also had higher levels of spongiosis, astrogliosis, and PrP deposition (Figs 6F and 9, S7 Fig and S2 Table) than those that displayed a more acute phenotype. None of the NBH(WT)^P2^, NBH(K_4_N)^P2^ or uninoculated age-matched control mice showed any indication of TSE disease by histopathology (Table 2 and S1C Table).

Collectively, these results indicated that upon second passage, infections initiated by ScBH(K_4_N)^RTQ^ and ScBH(WT)^RTQ^, but not their analogous NBH-seeded products, induced a serially transmissible prion disease. However, resulting diseases gave distinct clinical, biochemical and histopathological manifestations compared to classical 263K scrapie, indicating that both scrapie templating and mutation of the CLC lysines affect the properties of the synthetic prion strains produced in vitro from rPrP^Sen^ alone without cofactors, denaturants or physiologically implausible pHs.

## Discussion

### Prion propagation, clearance, and conversion of endogenous PrP^C^

Many, if not most, proteins are capable of assembling into self-propagating amyloid fibrils under certain conditions [36, 37]. A key question is whether such fibrils can be initiated and propagated under physiological conditions. Infectious prion aggregates must be able to survive in the host long enough to initiate formation of new aggregates at a rate that exceeds that of any clearance processes. This requires that the prions gain access to suitable pools of normal substrate molecules for recruitment into growing prion aggregates. In the case of prions made of PrP molecules, the interactions between incoming prions and PrP^C^ is likely to be constrained by the GPI-anchored membrane association and heavy glycosylation of most potential PrP^C^ substrate molecules, as well as the local availability of suitable cofactor molecules. If the infecting aggregate is *bona fide* PrP^Sc^ coming from another mammal that expresses wild-type PrP^C^, then the PrP^Sc^ is derived from endogenous PrP^C^ molecules and thus must have a conformation that can accommodate them. However, this might not be true of synthetic aggregates that are made *de novo* in *in vitro* reactions from recombinant PrP molecules that lack post-translational modifications. Such assemblies might be packed into conformations that, upon inoculation, are less able to accommodate new monomers that are membrane-bound or laden with bulky glycans. In such cases, the aggregate replication rate might be diminished, and unable to outpace clearance mechanisms and accumulate to toxic levels within the lifespan of the host. Our current data provide evidence that CLC mutations can not only affect the conformation of rPrP amyloids but also their ability to propagate utilizing endogenous wild-type PrP^C^ *in vivo* and cause pathology.

### Effects of scrapie templating

Here, and in other studies in which prions were made from recombinant PrP in physiologically compatible buffers but in the absence of natural cofactors, initial templating by PrP^Sc^ enhanced the infectivity of the rPrP fibrils [14, 26]. In our case, the ScBH(WT)^RTQ^ and ScBH(K_4_N)^RTQ^ products both were retained or propagated in the host and, on second passage, caused clinical prion disease. By contrast, the NBH(WT)^RTQ^ and NBH(K_4_N)^RTQ^ did not. The lack of infectivity in NBH(K_4_N)^RTQ^ was not solely due to a lack of seeding activity, because it propagated in serial RT-QuIC reactions and generated amyloid with a PK-resistant core, albeit one that differed somewhat from those of ScBH(WT)^RTQ^ and ScBH(K_4_N)^RTQ^ (Fig 1). Furthermore, the level of inoculated NBH(K_4_N)^RTQ^ seeding activity was 2–3 logs lower than that of the ScBH(WT)^RTQ^ and ScBH(K_4_N)^RTQ^ inocula (S1 Fig). However, seeding activity was detectable in NBH(K_4_N)^P1^ Tg7 mice 2 h post-inoculation at levels 1–2 logs lower than that of the ScBH(K_4_N)^P1^ Tg7 mice, yet the untemplated inocula did not amplify in vivo. Thus, either the untemplated conformation, which was distinct from the ScBH templated conformation [32], or the lower inherent seeding activity of the NBH(K_4_N)^RTQ^ product, may have rendered it non-pathogenic upon inoculation into rodents. Perhaps spontaneously arising (NBH-seeded) amyloids, even if partially PK-resistant, have a conformational architecture that is more easily targeted for destruction, or unable to accommodate endogenous PrP^C^ or cofactor molecules efficiently enough to outpace degradation. Our data suggest that this is less of a limitation when the rPrP^Res^ amyloid is templated by *bona fide* PrP^Sc^.

Nonetheless, scrapie templating alone, without cofactors, was insufficient to enable replication of fully pathogenic prions *in vitro* using recombinant WT, K_4_N or K_4_A PrP because neither ScBH(WT)^RTQ^, ScBH(K_4_N)^RTQ^, nor ScBH(K_4_A)^RTQ^ induced clinical disease on first passage. However, one hamster and 5 of the PrP^C^ overexpressing Tg7 mice inoculated with either ScBH(WT)^RTQ^ or ScBH(K_4_N)^RTQ^ accumulated high seeding activities (>10^8^ SD_50_/mg) (Fig 2; S1 Table). Thus, consistent with conclusions of previous studies [38–40], the accumulation of high levels of seeding activity was not necessarily coincident with clinical disease.

From a practical perspective, it is notable that we detected increases in seeding activity only when (*i*) certain ScBH-seeded RT-QuIC products were inoculated into transgenic mice that massively overexpress PrP^C^ or (*ii*) when unnatural mutant RT-QuIC products [ScBH(K_4_N)^RTQ^ or ScBH(K_4_A)^RTQ^] were inoculated into wild-type hamsters. The fact that the ScBH(WT)^RTQ^ products failed to propagate above discernable input levels in wild-type animals suggests that there is little, if any, biohazard associated with performing RT-QuIC assays under routine clinical circumstances in which WT rPrP substrates would be used by humans expressing normal amounts of PrP^C^. Indeed, in our bioassays 2.5 μg of RT-QuIC reaction product was inoculated directly into the brains of hamsters without any apparent pathological effect, making these RT-QuIC products at least 10^6^−10^9^-fold less pathogenic than *bona fide* 263K PrP^Sc^ (e.g. [1]) and PrP^Sc^-seeded products of brain homogenate-based PMCA reactions [41].

Interestingly, Kim et al induced clinical disease on first passage into hamsters inoculated with recombinant hamster 90–231 rPrP^Res^ generated in sonicated PMCA reactions containing 0.1% SDS and 0.1% Triton X100 [14]. In the present study, the ScBH(WT)^RTQ^ and ScBH(K_4_N)^RTQ^ products were shaken rather than sonicated and contained only 0.002% SDS. Whether sonication, the much higher detergent content, or the use of N-terminally truncated PrP in the Kim et al study was most responsible for the greater pathogenicity of the rPrP PMCA products is not clear. Notably, SDS has an anionic moiety that might ion-pair with the cationic CLC to facilitate tighter packing in this region. The hydrophobic tail of SDS could also play an important role, analogous to previously observed effects of detergents [30, 42] and lipids [31, 43] on the infectious properties and protease-resistant core of recombinant PrP amyloids.

### Effects of CLC mutations

Although scrapie seeding enhanced the pathogenicity of both the ScBH(WT)^RTQ^ and ScBH(K_4_N)^RTQ^ products, the mutant ScBH(K_4_N)^RTQ^ material produced more extensive seed amplification and prion pathogenesis on first passage into both hamsters and Tg7 mice (Tables 1 and 2 and S1A and S1B Table). On second passage in the mice, both of these infections caused clinical disease and extensive signs of prion pathogenesis, although the appearance of spongiosis and abnormal PrP was slower in the ScBH(WT)^RTQ^ mice (Fig 6). Given that single preparations of ScBH(WT)^RTQ^ and ScBH(K_4_N)^RTQ^ products were inoculated in the first passage, it remains possible that these differences observed *in vivo* might have been due to stochastic events during *in vitro* propagation that were not determined by the WT or K_4_N sequences specifically. However, the biochemical distinctions between the ScBH(WT)^RTQ^ and ScBH(K_4_N)^RTQ^ products shown in Fig 1B were consistently observed in three independent 3-round RT-QuIC reactions. Thus, at present the simplest explanation for the *in vivo* phenotypes is that they are linked in some way to the observed structural/conformational differences between the ScBH(WT)^RTQ^ and ScBH(K_4_N)^RTQ^ products. Nonetheless, it is difficult to exclude the possibility that the infectious agents produced in the RT-QuIC reactions were low-abundance rPrP species rather than the bulk of the PK-resistant forms that were detected by immunoblotting. Indeed, none of the combined RT-QuIC products that we have generated showed the same predominance of the 17-19-kDa band that is exhibited by PK- and PNGase F-treated 263K PrP^Sc^ (as shown in Fig 4). Thus, although our biochemical and bioassay data support the idea that the CLC residues and mutations thereof influence misfolding and transmissibility of PrP structures formed in the absence of cofactors, we cannot discriminate clearly between the possibilities that (i) the ScBH(WT)^RTQ^ and ScBH(K_4_N)^RTQ^ products are relatively uniform amyloids that are transmissible and ultimately pathogenic, but much less so than 263K PrP^Sc^, (ii) the products are mixtures of conformers, only a subset of which are transmissible and/or pathogenic but, again, less so than 263K PrP^Sc^ because we never detected clinical disease on first passage, and (iii) the products are the same as described in ii) but with interference of seed propagation *in vivo* by non-infectious or non-pathogenic conformers in the preparation.

In any case, our findings raise the question of why the K_4_N and K_4_A mutations promote the formation of more pathogenic scrapie-templated seeds *in vitro* in the absence of cofactors. One possibility is that neutralization of the cationic lysine sidechains relieves the need for charge compensation by anionic cofactors *in vitro* in order to achieve a conformation within the ~90–160 region [32, 33] that more readily enables the conversion and incorporation of PrP^C^ *in vivo*. However, these mutations in rPrP clearly did not allow for fully faithful propagation of the 263K PrP^Sc^ strain in RT-QuIC reactions. Thus, the latter must require other molecules or microenvironments that would be available *in vivo*. A key biochemical feature of the prions in the ScBH(K_4_N)^P1^ mice was their distinctive ~14-kDa PK-resistant core, a feature that was faithfully propagated in ScBH(K_4_N)^P2^ mice as well. These results suggest that multimers with only this 14-kDa C-terminal core can be infectious. However, the fact that the generation of these multimers was aided by inoculation of the ScBH(K_4_N)^RTQ^ products with N-terminally extended cores provides evidence that structure that is outside the 14-kDa C-terminal core influences the templating activity and transmissibility of synthetic PrP amyloids. Interestingly, the ScBH(WT)^P2^ mice generated an additional ~20 kDa PrP^Res^ species (Fig 8). This suggests that the ScBH(WT)^RTQ^ inoculum may have allowed the strain to diverge and generate both forms of PrP^Sc^, as has been described previously as “deformed templating” [44]. While the ~20 kDa species was only seen in the three ScBH(WT)^P2^ mice with clear clinical signs of TSE disease, we did not observe the ~20 kDa core in the clinical ScBH(K_4_N)^P2^ mice, indicating that this conformer is not required at immunoblot-detectable levels for the induction of clinical TSE disease.

Without PrP^Sc^ templating, neither the K_4_N nor the WT rPrP substrates formed aggregates that initiated seed amplification or pathology *in vivo*, despite the seeding activity observed in the NBH(K_4_N)^RTQ^ products. This confirms that recombinant PrP^C^ molecules do not readily adopt an infectious form in the absence of a PrP^Sc^ template to guide refolding and assembly. Altogether, the results of this study support the concept that tight folding in the vicinity of the CLC, which is near the N-termini of the PK-resistant and solvent-excluding cores of PrP^Sc^ protomers, is critical in the formation of pathogenic TSE prions. Moreover, the CLC lysines may inhibit the propagation of TSE prions in the absence of charge-compensating cofactors.

## Materials and methods

### Protein expression and purification

Recombinant hamster PrP^C^ and mutants were purified as previously described [45]. Briefly, BL21(DE3) Rosetta 2 *Escherichia coli* containing the pET41 vector (EMD, Billerica, MA, USA) with the PrP sequence (Syrian hamster residues 23–231; accession no. K02234, or the PrP sequence including the lysine mutations described in [32]) were grown in Luria broth medium in the presence of kanamycin and chloramphenicol. Protein expression was induced using the autoinduction system and purified using nickel-nitrilotriacetic acid superflow resin (Qiagen) with an ÄKTA pure chromatography system (GE Healthcare Life Sciences) as previously described [45]. The purified protein was extensively dialyzed into 10 mM sodium phosphate buffer (pH 5.8) and stored at −80°C. Protein concentration was determined by measuring absorbance at 280 nm.

### Inoculum

Scaled-up RT-QuIC reactions were performed as previously described [32]. Reaction mixtures were the same as for RT-QuIC with the exceptions of not including ThT and using the indicated substrates. Reactions were performed at a volume of 1 ml in a 1.5 ml tube in an Eppendorf Thermomixer-R at 700 rpm to generate RT-QuIC products. Reactions were run for 15 h with cycles of 60 s shaking and 60 s of rest throughout the incubation at 42°C. Tube-based 1000 uL RT-QuIC reactions were seeded with 2μL of a 10^−6^ 263K prion infected (263K) or normal Syrian Golden hamster (NBH) brain tissue dilutions to initiate templated conversion of the substrate and as a specificity control, respectively. Reactions were limited to an incubation of 15 hours to minimize the incidence of spontaneous conversion in the WT rPrP^Sen^ reactions. Following a 15 hour incubation one microliter of the first round reaction was used to seed a 1 ml second round reaction resulting in a 1:1000 fold dilution of the seed. After a 15 hour incubation we repeated the process by taking one microliter of the second round conversion product to seed a third and final round at an additional 1:1000 dilution of the seed. Three serial passages of the scaled up reactions were performed to achieve a final brain tissue dilution of at least 10^−12^.

### Brain homogenates

Brain homogenates were prepared as previously described [45] with the exception of being homogenized in 1X PBS and stored at -80°C. For RT-QuIC analysis BHs were serially diluted in 0.1% SDS (sodium dodecyl sulfate, Sigma)/N2 (Gibco)/PBS as previously reported [46].

### RT-QuIC

RT-QuIC was performed as previously described [45]. Briefly, for each reaction well 2ul of the indicated brain dilution was added to 98 μL of a reaction mixture resulting in a final concentration of 10 mM phosphate buffer (pH 7.4), 300 mM NaCl, 0.1 mg/mL Ha90-231 rPrP^Sen^, 10 μM ThT, and 1 mM ethylenediaminetetraacetic acid tetrasodium salt (EDTA). The reaction mix was loaded into each well of a black-walled 96-well plate with a clear bottom and reactions were seeded with 2 μL of the indicated dilution for a final reaction volume of 100 μL containing 0.002% SDS. Plates were sealed and incubated in a BMG FLUOstar plate reader for 30 h with cycles of 60 s shaking and 60 s of rest throughout the incubation at 55°C. ThT fluorescence measurements (450 ± 10 nm excitation and 480 ± 10 nm emission; bottom read) were taken every 45 min. Fluorescence reactions were judged to be positive or negative prior to 24 h as described previously [47]. Briefly, to compensate for differences between the plate readers, we averaged data from replicate wells and normalized to a percentage of the maximal fluorescence response of the instrument. The obtained values were plotted against the reaction times. Samples were judged to be positive if two or more wells crossed a 10% maximum ThT fluorescence threshold prior to the 24 h cutoff. Spearman-Kärber analysis was used to estimate the seeding dose at which 50% of the replicate wells became positive (SD_50_) [16, 48].

### Animal inoculations

Prior to inoculation procedures, animals were anesthetized with isoflurane for restraint and pain reduction. Animals were euthanized using carbon dioxide asphyxiation using standard methods recommended by American Veterinary Medical Association guidelines (https://www.avma.org/KB/Policies/Documants/euthanasia.pdf). Inoculations were performed as previously described [49]. RT-QuIC reaction products were diluted either 1:1 v/v or 1:10 v/v in inoculation buffer (phosphate-buffered balanced saline supplemented with 2% fetal bovine serum) and brain homogenates (10% in PBS) were diluted to the desired final concentration in inoculation buffer. Four- to 6-week-old Tg7 mice, overexpressing Syrian golden hamster (SGH) PrP ~5 fold [34], or SGH were inoculated intracerebrally (i.c.) with 50 μl of the inocula. Animals were observed daily for signs of neurological disease including unused nestlets, poor grooming, kyphosis, ataxia, wasting, delayed response to stimuli, and somnolence and were euthanized on confirmation of progressive neurological disease or at designated time points.

### Ethics statement

The animal experimental protocol was reviewed and approved by the Rocky Mountain Laboratories Animal Care and Use Committee (Animal Study Protocol 2014–005 and 2016–039). The Rocky Mountain Laboratories are fully accredited by the American Association for Laboratory Animal Care and this study was carried out in strict accordance with the recommendations in the Guide for the Care and Use of Laboratory Animals of the National Institutes of Health.

### Immunoblot analyses

RT-QuIC reaction products containing 0.1 mg/ml rPrP were treated with 15 μg/ml PK (CALBIOCHEM) at 37°C for 1 h. 20% Brain homogenates in PBS were treated with 50 μg/ml PK at 37°C for 1 h. Reactions were stopped by diluting in sample buffer containing a final concentration of 4 M urea, 4% SDS, 2% β-mercaptoethanol, 8% glycerol, 0.02% bromophenol blue and 50 mM Tris-HCl; pH 6.8. Sample were then analyzed using Western blot techniques as previously described [32]. Equal volumes of PK-treated reactions were run on 10% Bis-Tris NuPAGE gels (Invitrogen). Proteins were transferred to an Immobilon P membrane (Millipore) using the iBlot Gel Transfer System (Invitrogen). Immunoblotting was carried out using the following α-PrP antibodies: R20 (epitope: residues 218–231), R30 (epitope: 89–103) [50], 3F4 (Millipore; epitope: 109–112), mAB132 (epitope: 119–127) [51], and R18 (epitope: 143–156) [50, 52] as previously described [32, 33].

### Pathology and immunohistochemistry

As described previously [49], SGH or mice were euthanized, brains were removed, and the sagittal half contralateral to the site of inoculation was placed in 50 ml of 3.7% phosphate-buffered formalin for 3 to 5 days before dehydration and embedding in paraffin. New dissection tools were used for each dissection. Antigen retrieval was performed as previously described [49], followed by staining with rabbit monoclonal antibody (EP1802Y at a 1:6000 dilution) to PrP (Abcam). Astrocyte detection was performed by staining with polyclonal rabbit anti-glial fibrillary acidic protein (anti-GFAP; Dako). Slides were also stained for observation of overall pathology using a standard hematoxylin-eosin (H&E) protocol. All histopathology slides were analyzed by observers blinded to the animal inoculation groups. Histopathology was scored on a scale of 0 to 3 with 0 being no pathology in the entire brain and 3 being severe widespread pathology throughout the brain. Animals with a score above 0 in any of the three lesion types was marked positive for histopathology.

## Supporting information

S1 Fig End-point dilution RT-QuIC analysis of inocula. Representative RT-QuIC profiles of ScBH(WT)^RTQ^ product (top left), ScBH(K_4_N)^RTQ^ product (top right), NBH(WT)^RTQ^ product (bottom left), and NBH(K_4_N)^RTQ^ product (bottom right). Each sample was serially diluted down to 10^−9^ sample dilutions. Each trace is an average of four replicate wells. The SD_50_ per mg of inoculated RT-QuIC product is displayed above each panel.
(TIF)

S2 Fig End-point dilution RT-QuIC analysis of the brains from P1 animals. Representative end-point dilution RT-QuIC analysis of brain homogenates from hamsters (A [A465-1] & B [A458-2]) and Tg7 mice (C [B991-1] & D [B987-3]) inoculated with ScBH(WT)^RTQ^ (A & C) or ScBH(K_4_N)^RTQ^ (B & D). Hamsters were assayed at 10^−3^–10^−7^ brain tissue dilutions and Tg7 mice were assayed at 10^−4^–10^−9^ brain tissue dilutions. Brain tissue from non-inoculated (NBH) mice and hamsters were assayed at 10^−3^ dilutions as controls. Each trace is an average of four replicate wells. SD_50_ per mg of brain tissue are indicated above each panel.
(TIF)

S3 Fig Epitope mapping of newly formed PrP^Res^ in brains from ScBH(WT or K_4_N)^P1^ Tg7 mice. PrP antibodies R30 (A; epitope: 89–103), 3F4 (B; epitope: 109–112), mAB132 (C; epitope: 119–127), R18 (D; epitope: 143–156), and R20 (E; epitope: 218–231) were used. (F) Diagram outlining the location of antibody epitopes. The locations of the central lysine cluster (CLC), signal peptide (SP), glycosylphosphatidylinositol (GPI) anchor, and proteinase K (PK) cleavage site are also indicated.
(TIF)

S4 Fig Histopathology of brain samples from a control Tg7 mouse inoculated with ScBH. Brain regions affected in ScBH(PrP^Sen^)^P1^ as well as areas commonly affected by 263K scrapie are displayed. Slides were stained using a PrP antibody (EP1802Y), anti-GFAP antibody, or hematoxylin and eosin. Scale bar indicates 50 microns.
(TIF)

S5 Fig Histopathology of the hippocampus of Tg7 ScBH(WT or K_4_N)^P1^ mice and controls. Slides were stained using a PrP antibody (EP1802Y), GFAP antibody, or hematoxylin and eosin. Animal numbers are displayed below images. Red arrow heads denote PrP aggregates and black arrows denote vacuolization (spongiosis). Scale bar indicates 50 microns.
(TIF)

S6 Fig End-point dilution RT-QuIC analysis of the brains from P2 animals. Representative end-point dilution RT-QuIC analysis of one ScBH(WT)^P2^ brain homogenate (A [C204-2]) and one ScBH(K_4_N)^P2^ brain homogenate (B [C208-2]). Each sample was assayed down to 10^−10^ brain tissue dilutions. SD_50_ per mg of brain homogenate is displayed above each panel. The animal number used to inoculate the P2 animal assayed is displayed in parentheses. Each trace is an average of four replicate wells.
(TIF)

S7 Fig Histopathology of the hippocampus of Tg7 ScBH(WT or K_4_N)^P2^ and control mice. A comparison between ScBH(K_4_N)^P2^ Tg7 mice that had acute TSE disease at 143 dpi and ScBH(K_4_N)^P2^ Tg7 mice that had a prolonged clinical course out to 433 dpi is shown. Red arrow heads denote PrP aggregates and black arrow heads denote vacuolization (spongiosis). Slides were stained using a PrP antibody (EP1802Y), GFAP antibody, or hematoxylin and eosin. Animal numbers are displayed below images. Scale bar indicates 50 microns.
(TIF)

S1 Table Summary of all results for each animal. The table summarizes the individual animal data for A) Hamsters, B) Tg7 P1 mice, and C) Tg7 P2 mice.
(XLSX)

S2 Table Summary of histopathology results. The table describes the histopathology as it pertains to spongiosis, astrogliosis and PrP deposition for each ScBH animal.
(PDF)
