# Supplementary figures and images for: Role of the central lysine cluster and scrapie templating in the transmissibility of synthetic prion protein aggregates
Source: PLoS Pathog. 2017 Sep 14;13(9):e1006623. doi: 10.1371/journal.ppat.1006623 (PMC5614645; doi:10.1371/journal.ppat.1006623)

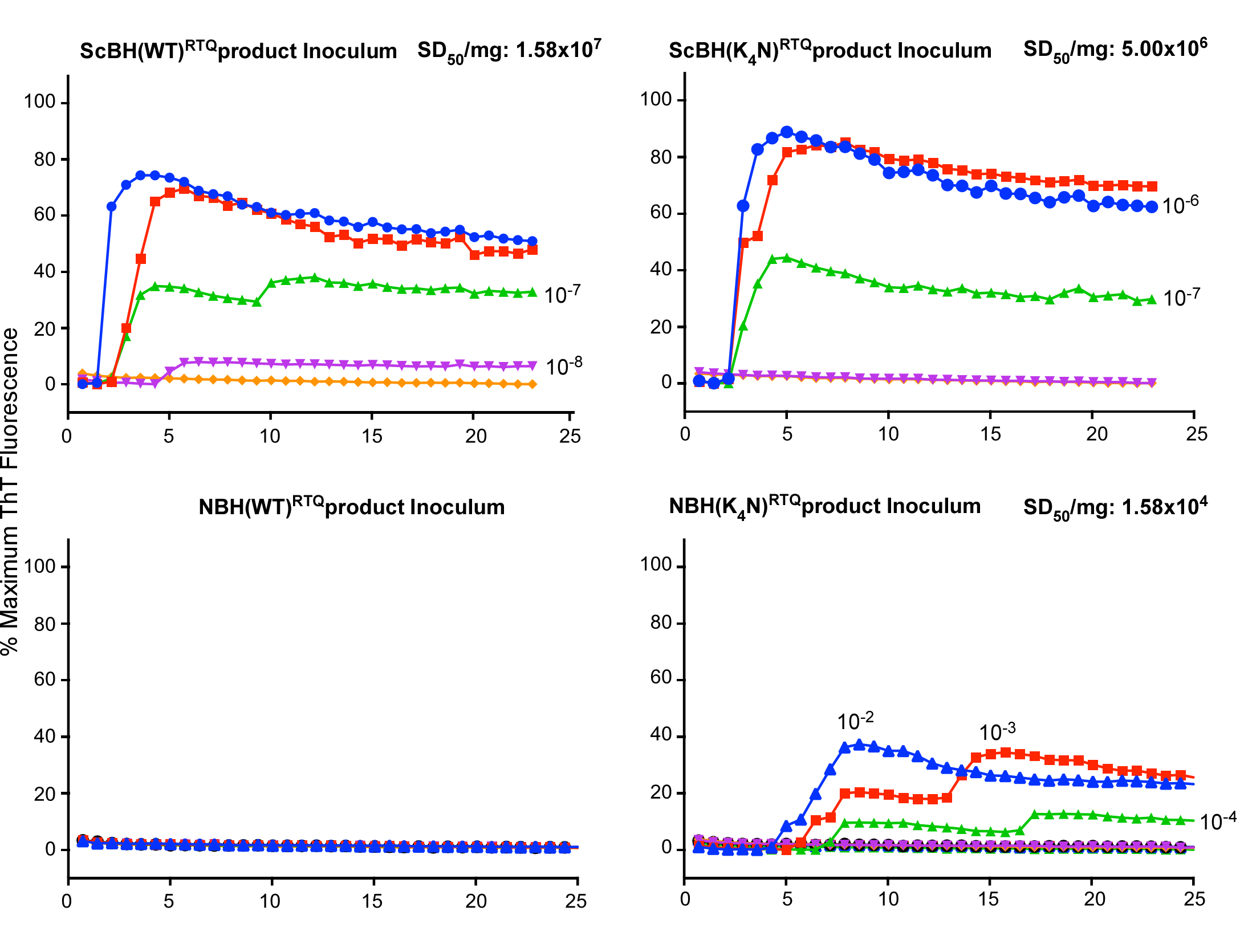

Supplement: S1 Fig — Representative RT-QuIC profiles of ScBH(WT)RTQ product (top left), ScBH(K4N)RTQ product (top right), NBH(WT)RTQ product (bottom left), and NBH(K4N)RTQ product (bottom right). Each sample was serially diluted down to 10−9 sample dilutions. Each trace is an average of four replicate wells. The SD50 per mg of inoculated RT-QuIC product is displayed above each panel. (TIF) [file ppat.1006623.s001.tif]

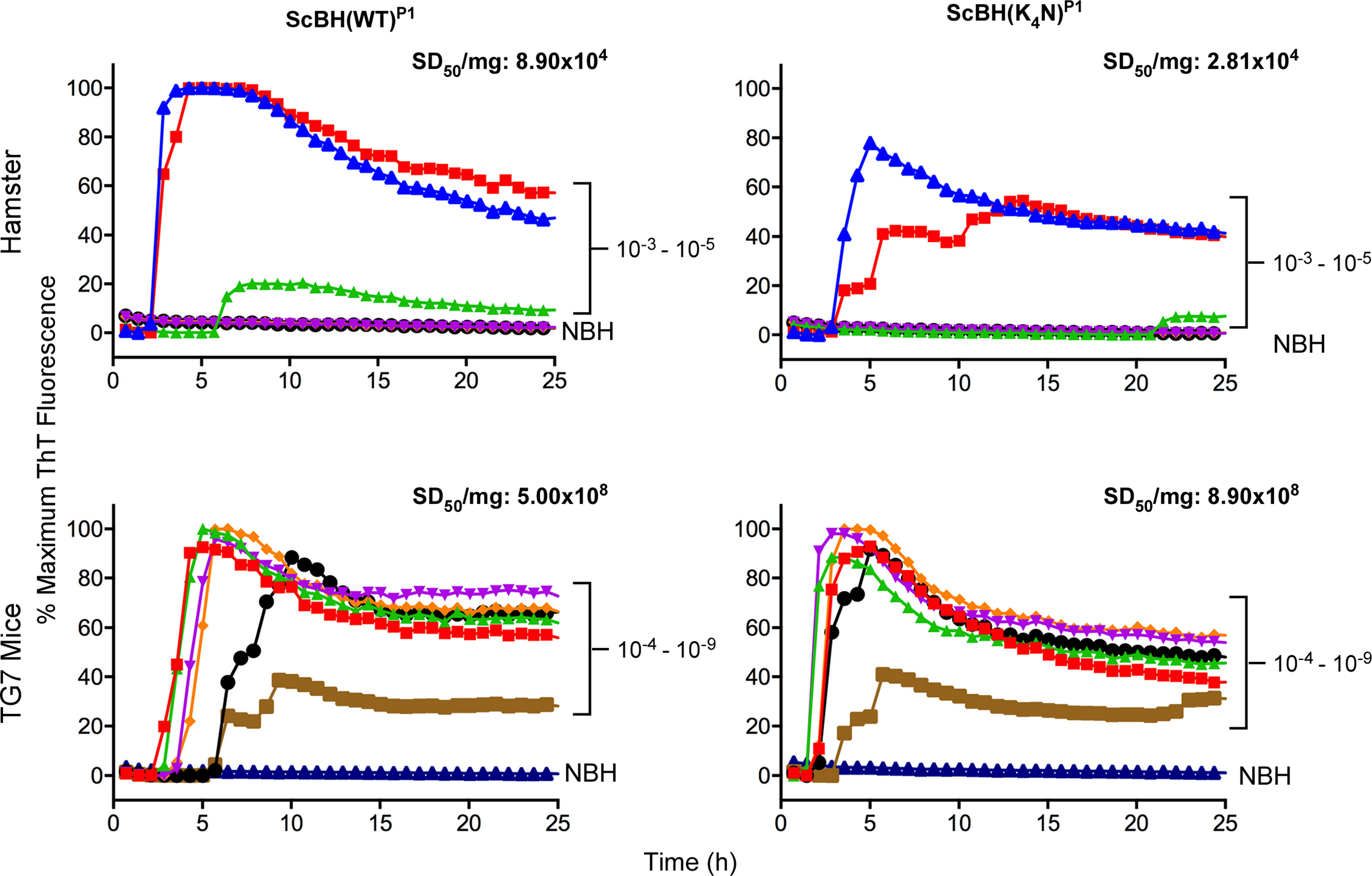

Supplement: S2 Fig — Representative end-point dilution RT-QuIC analysis of brain homogenates from hamsters (A [A465-1] & B [A458-2]) and Tg7 mice (C [B991-1] & D [B987-3]) inoculated with ScBH(WT)RTQ (A & C) or ScBH(K4N)RTQ (B & D). Hamsters were assayed at 10−3–10−7 brain tissue dilutions and Tg7 mice were assayed at 10−4–10−9 brain tissue dilutions. Brain tissue from non-inoculated (NBH) mice and hamsters were assayed at 10−3 dilutions as controls. Each trace is an average of four replicate wells. SD50 per mg of brain tissue are indicated above each panel. (TIF) [file ppat.1006623.s002.tif]

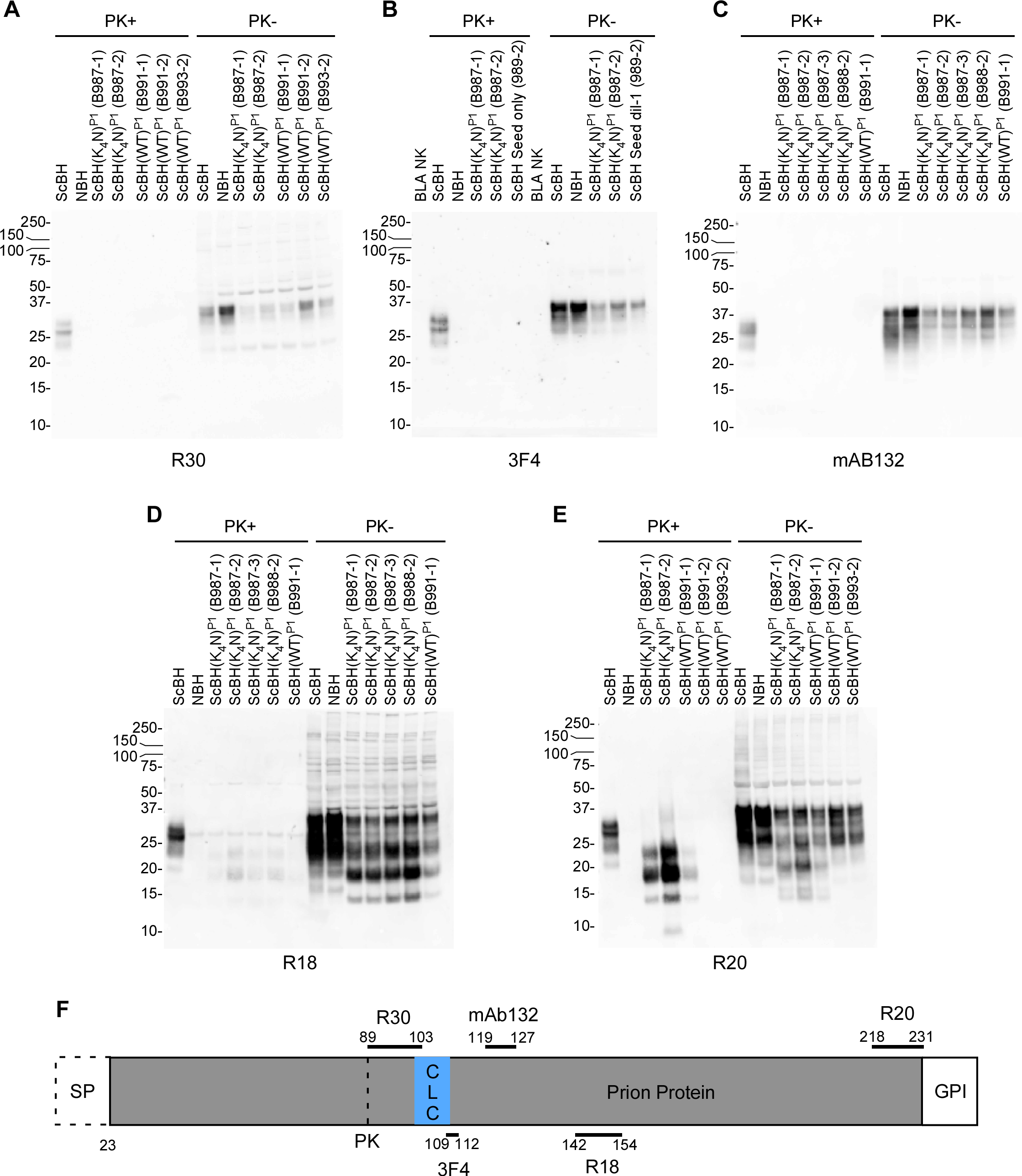

Supplement: S3 Fig — PrP antibodies R30 (A; epitope: 89–103), 3F4 (B; epitope: 109–112), mAB132 (C; epitope: 119–127), R18 (D; epitope: 143–156), and R20 (E; epitope: 218–231) were used. (F) Diagram outlining the location of antibody epitopes. The locations of the central lysine cluster (CLC), signal peptide (SP), glycosylphosphatidylinositol (GPI) anchor, and proteinase K (PK) cleavage site are also indicated. (TIF) [file ppat.1006623.s003.tif]

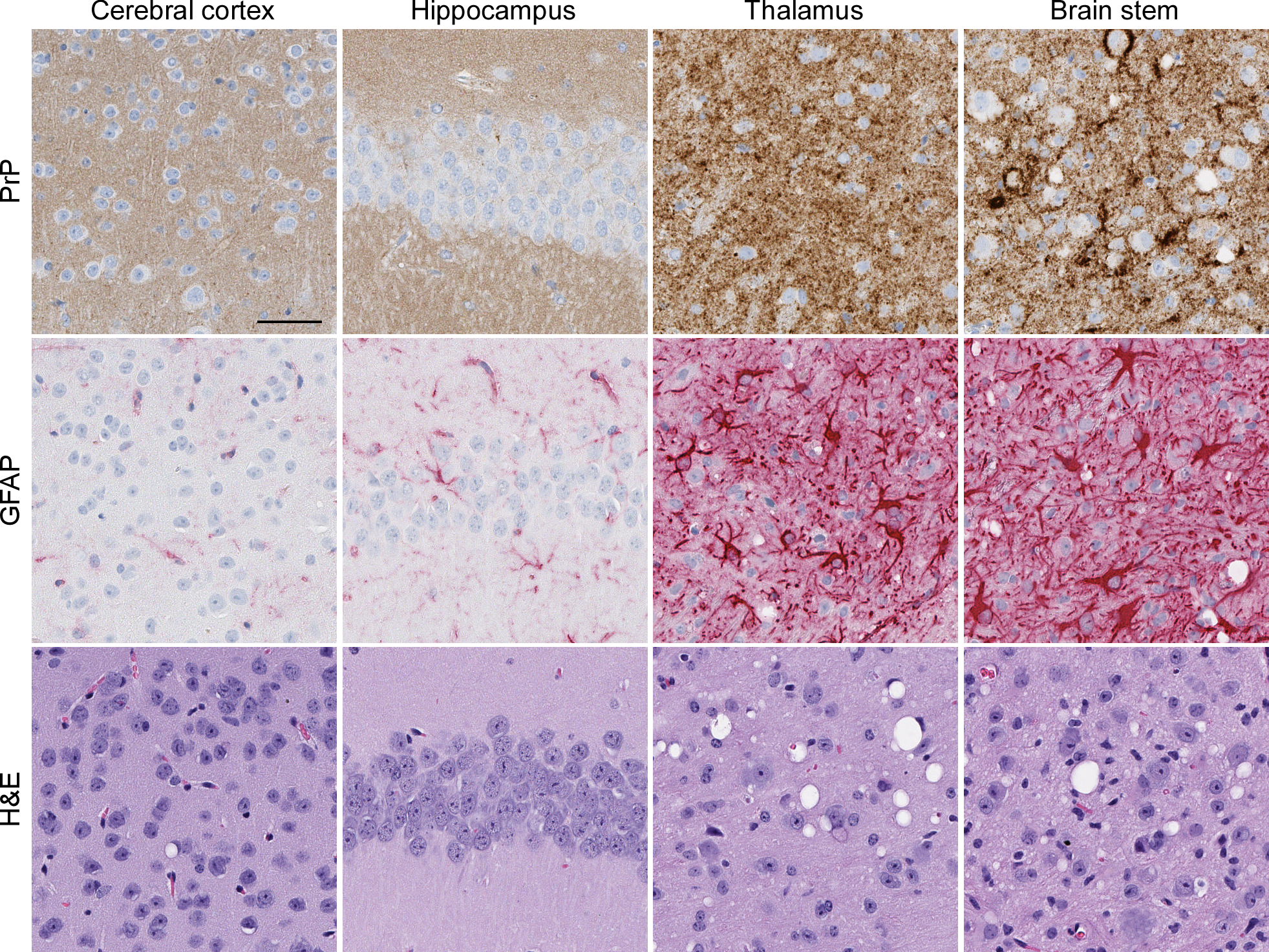

Supplement: S4 Fig — Brain regions affected in ScBH(PrPSen)P1 as well as areas commonly affected by 263K scrapie are displayed. Slides were stained using a PrP antibody (EP1802Y), anti-GFAP antibody, or hematoxylin and eosin. Scale bar indicates 50 microns. (TIF) [file ppat.1006623.s004.tif]

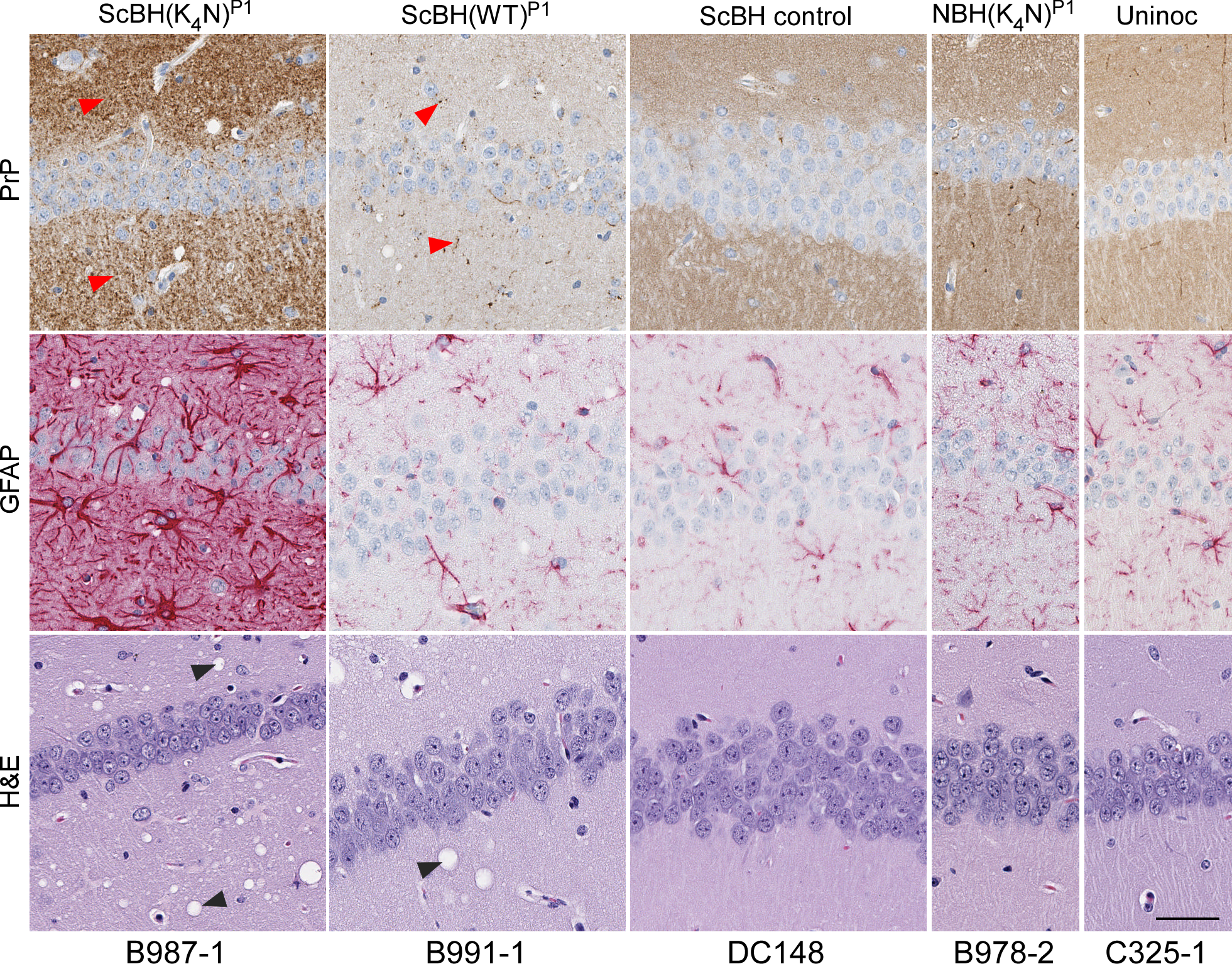

Supplement: S5 Fig — Slides were stained using a PrP antibody (EP1802Y), GFAP antibody, or hematoxylin and eosin. Animal numbers are displayed below images. Red arrow heads denote PrP aggregates and black arrows denote vacuolization (spongiosis). Scale bar indicates 50 microns. (TIF) [file ppat.1006623.s005.tif]

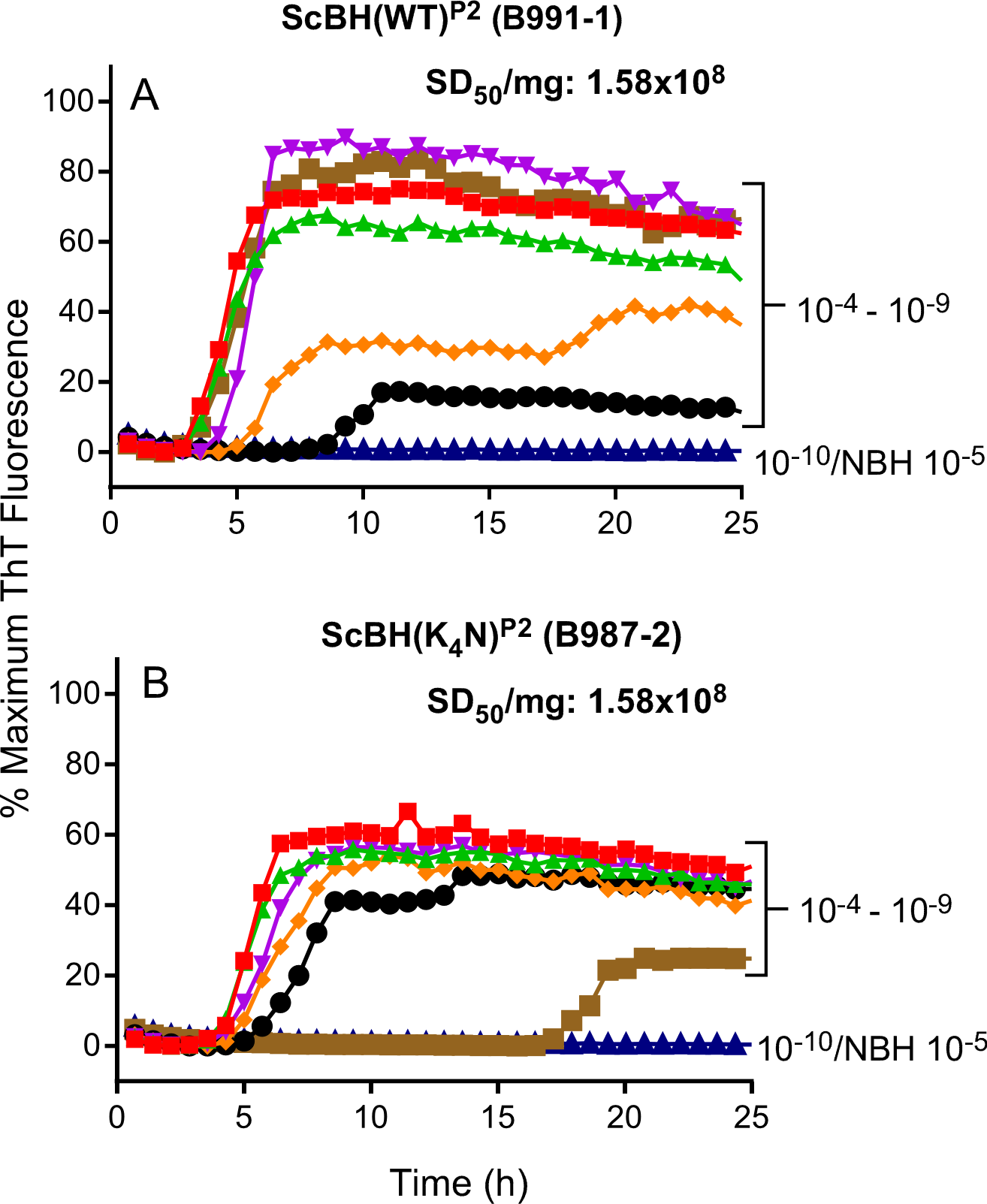

Supplement: S6 Fig — Representative end-point dilution RT-QuIC analysis of one ScBH(WT)P2 brain homogenate (A [C204-2]) and one ScBH(K4N)P2 brain homogenate (B [C208-2]). Each sample was assayed down to 10−10 brain tissue dilutions. SD50 per mg of brain homogenate is displayed above each panel. The animal number used to inoculate the P2 animal assayed is displayed in parentheses. Each trace is an average of four replicate wells. (TIF) [file ppat.1006623.s006.tif]

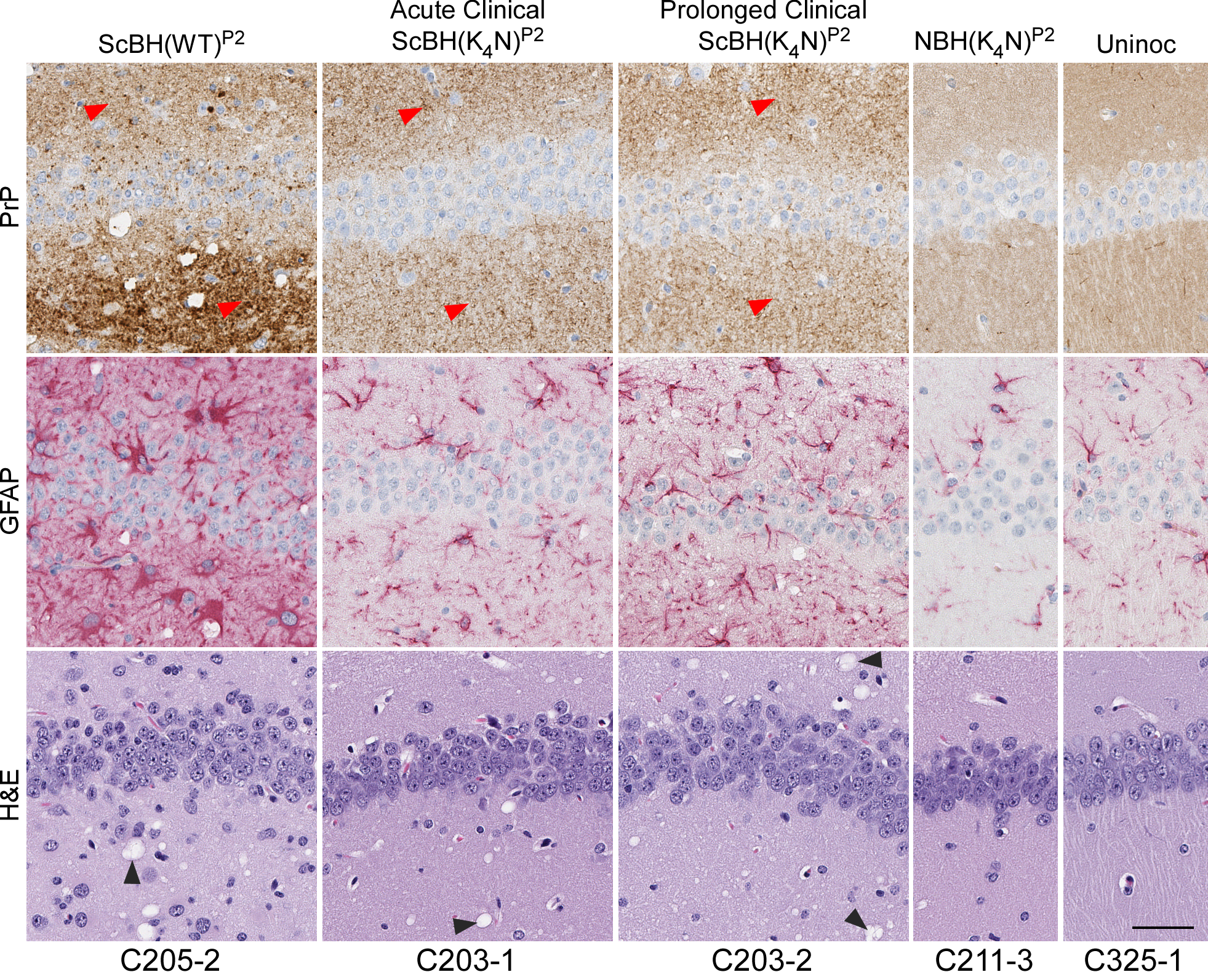

Supplement: S7 Fig — A comparison between ScBH(K4N)P2 Tg7 mice that had acute TSE disease at 143 dpi and ScBH(K4N)P2 Tg7 mice that had a prolonged clinical course out to 433 dpi is shown. Red arrow heads denote PrP aggregates and black arrow heads denote vacuolization (spongiosis). Slides were stained using a PrP antibody (EP1802Y), GFAP antibody, or hematoxylin and eosin. Animal numbers are displayed below images. Scale bar indicates 50 microns. (TIF) [file ppat.1006623.s007.tif]
