# Supplementary material for: Role of the central lysine cluster and scrapie templating in the transmissibility of synthetic prion protein aggregates
Source: PLoS Pathog. 2017 Sep 14;13(9):e1006623. doi: 10.1371/journal.ppat.1006623 (PMC5614645; doi:10.1371/journal.ppat.1006623)
Supplement: S2 Table — The table describes the histopathology as it pertains to spongiosis, astrogliosis and PrP deposition for each ScBH animal. (PDF) [file ppat.1006623.s009.pdf]

S2 Table: Summary of histopathology results

	Animal #	DPI	Spongiosis	Astrogliosis	PrP deposition
ScBH(K_4N)P^1 Hamsters	A457-2	496	negative	negative	negative
	A457-1	496	negative	negative	weak in the lateral ventricle along ependymal cells and around the parenchyma near the ventricle
	A459-1	624	weak, focal (CC and HC)	weak around PrP deposits	weak, widespread diffuse, and subtle focal vacuolation in the CC
	A459-2	652	weak, focal (CC)	negative	weak to moderate, widespread deposition (CC and HC)
ScBH(WT)P^1 Hamsters	A464-1	496	negative	negative	negative
	A464-2	496	negative	negative	negative
	A463-1	624	negative	negative	negative
	A463-2	652	negative	negative	negative
ScBH(K_4A)P^1 Hamsters	A519-1	467	negative	negative	negative
	A519-2	467	negative	negative	negative
	A531-1	561	negative	negative	weak, diffuse deposition in the CC
	A531-2	561	negative	negative	weak, focal plaque-like deposits lining the meninges and ependymal cells
	A525-2	611	negative	negative	negative
ScBH(K_4N)P^1 Mice	B987-1	392	moderate to severe, widespread (CC, SP, PO, and HC)	severe around lesions	weak small punctate and granular aggregates (HT and ST) as well as diffuse punctate deposition (HC, CC and ST)
	B987-2	392	moderate to severe, widespread (CC, SP, PO, and HC)	severe around lesions	weak small punctate and granular aggregates (HT and ST) as well as diffuse punctate deposition (HC, CC and ST)
	B987-3	492	moderate to severe, widespread	severe around lesions	weak small punctate and granular aggregates (HT and ST) as well as diffuse punctate deposition (HC, CC and ST)
	B988-2	492	moderate to severe, widespread (CC, SP, PO, CB, and HC)	severe around lesions	weak small punctate and granular aggregates (HT and ST) as well as diffuse punctate deposition (HC, CC, CB and ST)
ScBH(WT)P^1 Mice	B993-1	104	negative	negative	negative
	B994-1	392	negative	negative	negative
	B994-2	392	negative	weak	negative
	B991-1	492	weak (TH)	weak (TH)	weak (TH, CC)
	B991-2	492	negative	weak	negative
	B993-2	492	negative	weak	negative
ScBH(K_4N)^{P2} Mice	C202-1	143	weak (TH and CC)	negative	weak, widespread punctate deposition, especially in CC
	C203-1	143	weak (TH and CC)	moderate (CC, HC and TH)	weak, widespread punctate deposition, especially in CC
	C206-1	143	weak (TH and CC)	moderate (CC, HC and TH)	weak, widespread punctate deposition, especially in CC, SP, and ST
	C206-2	143	weak to moderate (TH , HC, and CC)	moderate (CC, HC and TH)	weak, widespread punctate deposition, especially in CC, SP, and ST
	C207-3	143	weak	moderate to severe (CC, SP, and TH)	weak, widespread punctate deposition, especially in CC, SP, and HC
	C208-2	143	weak to moderate (TH , HC, and SP)	moderate (HC, SP, and TH)	moderate, widespread punctate deposition, especially in FB, SP, and HC
	C209-1	143	weak to moderate (TH and HC)	moderate to severe (HC and TH)	weak, widespread punctate PrP deposition, especially in CC and HC
	C203-2	433	severe, widespread and focal (HC)	moderate to severe, widespread and especially in HC	severe, widespread diffuse deposition especially in HC, CC, CL and FB
	C203-3	433	severe, widespread and focal (HC)	moderate to severe, widespread and especially in HC	severe, widespread deposition especially in HC and CC
	C209-3	433	severe, widespread and focal (HC)	moderate to severe, widespread and especially in HC	severe, widespread deposition especially in HC and CC
ScBH(WT)^{P2} Mice	C204-1	143	weak	weak to moderate (CC, TH, and HC)	weak, widespread punctate deposition in many GM regions
	C204-2	143	weak	weak to moderate (CC, TH, and HC)	weak, widespread punctate deposition in many GM regions. Focal deposits in CB and MB
	C205-1	245	moderate to severe, widespread (TH, HT, CC)	severe, around PrP deposits and vacuoles	moderate, diffuse and small aggregates (BS, CB, TH, HT, PO, CC)
	C205-2	251	moderate to severe, widespread (TH, HC, HT, CC)	severe, around PrP deposits and vacuoles	severe, diffuse and small aggregates (BS, CB, TH, HT, HC, CC, PO, SP)
	C205-3	251	moderate to severe, widespread (TH, HC, HT, CC)	severe, around PrP deposits and vacuoles	moderate to severe, diffuse and small aggregates (BS, CB, TH, HT, HC, CC)

CC: cerebral cortex; SP: septum; PO: preoptic area; HC: hippocampus; HT: hypothalamus; ST: striatum; TH: thalamus; CB: cerebellum; FB: forebrain; CL: colliculus; GM: gray matter; MB: midbrain; BS: brain stem
